# Activation of mitochondrial CPS1 promotes dormant ovarian follicle activation via arginine elevation and the mTORC1 pathway

**DOI:** 10.3389/fcell.2026.1787020

**Published:** 2026-04-23

**Authors:** Stine Bundgaard Birkebæk, Mahboobeh Amoushahi, Karin Lykke-Hartmann

**Affiliations:** 1 Department of Biomedicine, Aarhus University, Aarhus, Denmark; 2 Department of Clinical Genetics, Aarhus University Hospital, Aarhus, Denmark

**Keywords:** arginine, CPS1, mTOR pathway, NCG, primordial follicle activation

## Abstract

**Background:**

Carbamoyl phosphate synthetase 1 (CPS1) is a mitochondrial ligase essential for urea production, catalyzing the formation of carbamoyl phosphate from ammonia in a reaction requiring the allosteric activator N acetyl L glutamate (NAG). Through the urea cycle, CPS1 activity ultimately contributes to arginine synthesis, a known activator of mTOR. The NAG analogue N-carbamoyl-L-glutamate (NCG) has been shown to promote follicle development in several species, potentially via mTOR signaling; however, its specific role in folliculogenesis remains unclear.

**Methods:**

Cultured murine ovaries and human ovarian cortical tissue fragments were treated with NCG to assess its effects on primordial follicle activation. Human granulosa like KGN cells were used to evaluate NCG induced changes in proliferation, arginine levels, and mTOR pathway activation. Mechanistic studies examined the interaction between CASTOR1 and GATOR2 in response to NCG supplementation.

**Results:**

NCG mediated activation of CPS1 promoted the awakening of dormant (primordial) follicles in both mouse ovaries and human ovarian cortical tissues. In KGN cells, NCG enhanced cell proliferation and increased intracellular arginine levels, accompanied by an elevated pS6K/S6K ratio, consistent with mTORC1 activation. Mechanistically, NCG induced arginine accumulation reduced CASTOR1 binding to its inhibitor GATOR2, thereby facilitating S6K phosphorylation and activation of the mTORC1 signaling cascade. siRNA-mediated knockdown reduced CPS1 protein levels, which eliminated NCG’s effect on arginine levels and the phosphorylation of S6K.

**Conclusion:**

NCG activates CPS1 to elevate arginine levels, relieve CASTOR1 mediated inhibition of GATOR2, and stimulate mTORC1 signaling. This pathway promotes primordial follicle activation and supports follicle development, highlighting NCG as a potential modulator of ovarian folliculogenesis.

## Introduction

The ovary is the central female reproductive organ, housing a finite reserve of oocytes essential for fertility. This reserve primarily consists of dormant primordial follicles, which are oocytes surrounded by flattened pregranulosa cells. At puberty, the average number of primordial follicles is approximately 400,000 (Faddy and Gosden, 1996; Rimon-Dahari et al., 2016; Sun et al., 2017). These follicles can remain quiescent for decades before activation. During the reproductive lifespan, a subset of primordial follicles is recruited monthly for growth through folliculogenesis, a process in which follicles progress through distinct developmental stages. Ultimately, one dominant follicle ovulates, while the remaining activated follicles undergo atresia (Faddy and Gosden, 1996; Rimon-Dahari et al., 2016; Sun et al., 2017). Primordial follicle activation is a tightly regulated process involving a balance of activating and inhibitory signals. This regulation occurs through paracrine communication between the oocyte and surrounding granulosa cells, as well as endocrine signaling via the hypothalamic–pituitary–ovarian axis (Chen et al., 2020). Among the intracellular pathways implicated in this process, the phosphatidylinositol 3′-kinase (PI3K)/protein kinase B (AKT) and mammalian target of rapamycin complex 1 (mTORC1) signaling cascades play pivotal roles (Makker et al., 2014). The PI3K-AKT pathway in the oocyte is initiated when kit ligand secreted by pregranulosa cells binds to the kit receptor on the oocyte surface. This interaction activates PI3K, leading to AKT phosphorylation by 3-phosphoinositide-dependent kinase 1 (PDK1). Activated AKT phosphorylates forkhead box O3a (FOXO3a), resulting in its nuclear export and subsequent follicle activation (De Felici and Klinger, 2021; John et al., 2008; Makker et al., 2014; Reddy et al., 2008).

The mTORC1 complex, which regulates cell growth and metabolism, is composed of multiple proteins, including Raptor, a key component required for mTORC1 activity. mTORC1 signaling is modulated by growth factors, amino acids, and cellular stress through the tuberous sclerosis complex (TSC1-TSC2). Phosphorylated AKT inhibits TSC1-TSC2, releasing Rheb, which recruits mTORC1 to the lysosomal surface and activates its kinase function. Activated mTORC1 phosphorylates S6 kinase 1 (S6K1), which subsequently phosphorylates ribosomal protein S6 (rpS6), promoting protein synthesis and follicle activation (Ghosh and Kapur, 2017; Takahara et al., 2020; Adhikari et al., 2009; Adhikari et al., 2010) De Felici & Klinger, 2021 (Makker et al., 2014). In addition to cell signaling, mitochondria play a critical role in follicle development by providing energy and protecting DNA from reactive oxygen species (ROS). Mitochondrial dysfunction has been linked to female infertility (Almansa-Ordonez et al., 2020; Avila et al., 2016; Wang et al., 2017). Consistent with this, transcripts encoding free radical scavengers have been detected in granulosa cells from primordial and primary follicles, suggesting mitochondrial involvement in maintaining oocyte competence (Ernst et al., 2018). Several mitochondrial proteins have been identified in human primordial and primary follicles (Ernst et al., 2018; Ernst et al., 2017), including carbamoyl phosphate synthetase I (CPS1), a key enzyme catalyzing the first and rate-limiting step of the urea cycle (Barmore et al., 2024). CPS1 activity requires binding of the allosteric activator N-acetyl-L-glutamate (NAG), which induces its active conformation (Caldovic et al., 2010). NAG deficiency disrupts the urea cycle, causing hyperammonemia; treatment involves N-carbamoyl-L-glutamate (NCG), a pharmaceutical NAG analogue (Tuchman et al., 2008).

Interestingly, NCG supplementation has been shown to influence ovarian follicle development. Dietary NCG increased follicle numbers and plasma amino acids in chickens (Ma et al., 2020) and enhanced large follicle formation in yaks (Zhou et al., 2022). These findings suggest that CPS1 activation by NCG may promote follicle maturation. However, the molecular mechanism underlying NCG’s effect on follicle activation remains unclear. Given that arginine—a urea cycle product—activates mTORC1 signaling in other tissues (Yao et al., 2008; Carroll et al., 2016), we hypothesize that NCG-mediated CPS1 activation elevates arginine levels, thereby stimulating mTORC1 signaling in primordial follicles. Arginine-dependent mTORC1 activation involves Rag GTPases, GATOR complexes, and lysosomal sensors such as SLC38A9, which facilitate mTORC1 recruitment to the lysosome and subsequent activation by Rheb (Wolfson et al., 2017; Chantranupong et al., 2016; Gai et al., 2016; Shen and Sabatini, 2018; Zoncu et al., 2011; Takahara et al., 2020).

This study investigates whether NCG supplementation activates primordial follicles via mTORC1 signaling. Using *in vitro* murine ovaries, *ex vivo* human ovarian tissue, and KGN cell models, we assess follicle activation and mTORC1 pathway engagement following NCG supplementation. Our findings provide mechanistic insight into NCG’s role in follicle maturation and its potential application in reproductive biology and animal husbandry.

## Materials and methods

### Animals

For C57BL X CBA F1 mice, female C57BL/6JRj mice and male CBA/JRj mice were crossbred. The mice were housed under a 12-h light/dark cycle with food and water *ad libitum* at the Department of Biomedicine at Aarhus University. All animal handling and experiments were approved by the Ethics Committee for the use of laboratory animals at Aarhus University (permit number: 2020-15-0201-00757 to KLH). We confirm that the study reported is in accordance with ARRIVE guidelines.

### *In vitro* mouse ovarian culture

Ovaries were collected from 7–8 postnatal F1 female mice and isolated from the surrounding tissue in culture medium containing α-minimal essential medium (α-MEM) (Gibco, 22571–020) supplemented with 10% foetal bovine serum (FBS) (Biowest, S18860–100), 1% insulin-transferrin-selenium (100 mg/mL) (ITS) (Thermo Fisher Scientific, 41400045), 1% penicillin‒streptomycin (100 mg/mL and 50 ng/mL) (P/S) (Thermo Fisher Scientific, 5140122) and 100 mIU/mL recombinant follicle stimulating hormone (FSH) (Merck, GONAL-F75IE-IU). The ovaries were randomly divided into control and experimental groups and transferred to inserts (Greiner Bio-one, 662641) placed in 24-well plates (Corning, 3526) (Amoushahi and Lykke-Hartmann, 2021; Ataei-Nazari et al., 2022; Madsen et al., 2023; Madsen et al., 2024). The ovaries were cultured in media consisting of α-MEM supplemented with 10% FBS, 1% ITS, 1% P/S, 100 mIU/mL FSH (Merck, GONAL-F75IE-IU), sodium selenite (10 ng/mL) (SS) (Sigma‒Aldrich, L-2654) and NCG (5–50 µM) (1 mg/mL, dissolved in mQH_2_O) (Sigma‒Aldrich, C4375-10G) in the experimental group or mQH_2_O in the control group. The ovaries were cultured for 96 h at 37 °C with an atmosphere of 95% humidity and 5% CO_2_, and half of the medium was changed every other day.

### *Ex vivo* human ovarian tissue culture

The human ovarian biopsies were donated from women at Aarhus University Hospital undergoing oophorectomy prior to gonadotoxic treatment of malignant disease (unrelated to the ovary) or prophylactic ovary removal to reduce the risk of developing ovarian cancer (written informed consent was obtained from women under a protocol approved by the Ethics Committee of the Faculty of Aarhus University, permit number: 2020–15-02-01–00757), confirming that all experiments were performed in accordance with relevant guidelines and regulations. The cortex of the ovarian tissue was separated from the medulla in medium containing L_15_ (Gibco, 3145–029) supplemented with 10% human serum albumin (HSA) (CSL Behring, 109697), after which the cortex was cleaned by gentle strokes along the tissue. The cleaned tissue was cut into 1 × 1 mm pieces and placed in 96-well plates (Corning, 3894) with culture medium consisting of α-MEM supplemented with 10% HSA, 1% ITS, 1% P/S (100 mg/mL and 50 ng/mL), FSH (300 mIU/mL), SS (10 ng/mL) and 30 µM NCG (1 mg/mL, dissolved in mQH_2_O) (Sigma‒Aldrich, C4375-10G) or mQH_2_O in the control group. The tissue was cultured for 14 days at 37 °C with an atmosphere of 95% humidity and 5% CO_2_, and half of the medium was changed every other day (Madsen et al., 2024).

### Tissue processing and staining

For histological analysis, cultured ovaries/tissue were fixed overnight in 4% paraformaldehyde (PFA) (Thermo Fisher Scientific, J199943-K2), dehydrated in ethanol (70%, 80%, 90%, 100%), cleared in xylene (VWR chemicals, 28973.363), infiltrated and embedded in paraffin (Leica Biosystems, 39601006) at 60 °C. The embedded tissue was sectioned at 4 µm using a microtome (SLEE medical GmbH, CUT 6062) and mounted onto glass slides for haematoxylin (Sigma‒Aldrich, MHS32-1 L) and eosin (Merck, 17372–87-) (H&E) staining. Here, the embedded sections were deparaffinized at 60 °C for 30 min and then placed in xylene (for 15 min twice). The sections were subsequently rehydrated in ethanol (3% × 100%, 96%, 70%) for 2 min each, followed by haematoxylin and eosin staining. Finally, the sections were dehydrated in ethanol (96%, 2% × 100%), cleared in xylene and mounted (Sigma‒Aldrich, 3989).

### Histological analysis

For determination of the follicle distribution in the ovaries/tissue, follicles in every 5^th^ section of H&E-stained ovaries were classified and counted as follows: primordial follicles as oocytes surrounded by flattened pregranulosa cells, primary follicles as oocytes surrounded by one layer of cuboidal granulosa cells, and secondary follicles as oocytes surrounded by more than one layer of cuboidal granulosa cells. Follicles consisting of an oocyte surrounded by one layer of a mixture of flattened pregranulosa cells and cuboidal granulosa cells were classified as intermediate follicles and were counted as primary follicles. Only follicles with clear nuclei were counted to prevent recounting. An inverted microscope (Leica, DMI4000 B) was used for the histological analyses (Amoushahi and Lykke-Hartmann, 2021; Ataei-Nazari et al., 2022; Madsen et al., 2023; Madsen et al., 2024).

### Immunofluorescence of mouse ovaries

For localization of the intracellular proteins in cultured mouse ovaries, immunofluorescence (IF) was performed. After 96 h of *in vitro* culture, the ovaries were fixed in 4% PFA (Thermo Fisher Scientific, J199943-K2) overnight, dehydrated in ethanol (70%, 80%, 90%, 100%), cleared in xylene (VWR chemicals, 28973.363), infiltrated and embedded in paraffin (Leica Biosystems, 39601006) at 60 °C. The embedded tissue was sectioned at 4 µm using a microtome (SLEE Medical GmbH, CUT 6062), and three representative sections from each ovary were mounted onto glass slides for IF staining. The sections were deparaffinized, rehydrated, and subjected to antigen retrieval with 0.1 M sodium citrate buffer, followed by permeabilization with Triton 100X (0.5% in PBS) for 10 min. The sections were blocked with 10% donkey serum (Chemicon, 220808) supplemented with 0.04 g/mL BSA (Clone, SH30574.02). Finally, the samples were incubated with a primary antibody against CPS1 (1:200) (Abcam, 45956) overnight. The following day, the sections were incubated with the secondary antibody ALEXA Fluor 488 donkey-anti-rabbit (1:300) (Thermo Fisher Scientific, A21206) for 1 h, counterstained with DAPI (1:700) (1 μg/mL in 10% donkey serum in PBS) (Thermo Fisher Scientific, 62248) and mounted with fluorescence mounting medium (DAKO, S3023). Images of the ovarian sections were captured with an LSM800 laser-scanning confocal microscope objective (Amoushahi and Lykke-Hartmann, 2021; Ataei-Nazari et al., 2022; Madsen et al., 2023; Madsen et al., 2024).

### TUNEL analysis

For quantification of apoptosis in 96-h cultured mouse ovaries, a TUNEL assay was performed using an *in situ* Cell Death Detection Kit (Roche, 11684795910) as described by the manufacturer, with few adjustments. In brief, the embedded ovaries were sectioned at 5 μm, and three representative sections from the centre of the ovaries were mounted on glass slides. The sections were deparaffinized at 60 °C and xylene (VWR chemicals, 28973.363), rehydrated in ethanol (100%, 96%, 70%) and then placed in water. For permeabilization of the sections, 20 μg/mL proteinase K (Thermo Fisher Scientific, EO0491) was used, followed by labelling with TUNEL reaction mix for 2 hours in a humidity chamber at 37 °C. The TUNEL reaction enzyme was excluded from the negative controls. Finally, the sections were counterstained with DAPI (1:700) (1 μL/mL) (Thermo Fisher Scientific, 62248) and mounted with fluorescent mounting medium (DAKO, S3023). To analyse and calculate the total number of apoptotic cells in the representative ovarian sections, fluorescence imaging was used (Molecular Devices, PICO imageXpress) (Amoushahi and Lykke-Hartmann, 2021; Ataei-Nazari et al., 2022; Madsen et al., 2023; Madsen et al., 2024).

### Western blotting analysis of mouse ovaries

After the end of 6 hours culture period, the ovaries were rinsed in phosphate-buffered saline (PBS) (Biowest, X0515-500), pooled and lysed in lysis buffer (1% SDS, 1 mM Na3VO4, 10 mM Tris, pH = 7.4) supplemented with 1% protease and phosphatase inhibitors (Thermo Fisher Scientific, 1861281). The protein concentration was measured by the Lowry method (Bio-Rad, 5000112) with BSA (HyClone, SH30574.02) as a standard (blank, 1.52, 0.76, 0.38, 0.19, 0.095 mg/mL), after which 25 µg or 50 µg of protein was used per lane. The samples were heated at 95 °C for 2 min before electrophoresis on SDS‒PAGE gels (Invitrogen, XV04120PK20), followed by transfer onto PVDF membranes (Thermo Fisher Scientific, 88518) for 1.5 h at 0.01 A. The membrane was blocked with Tris-buffered saline (pH = 7.6) (TBS) supplemented with 0.1% Tween-20% and 5% BSA (HyClone, SH30574.02) or 5% nonfat dry milk (MilliPore, 70166-500G) for 1 h at room temperature. The membranes were subsequently incubated overnight with primary antibodies against CPS1 (1:1000) (Abcam, ab45956), phosphorylated S6K (Thr389) (1:1000) (Cell Signaling Technology, 9205), total S6K (1:1000) (Cell Signaling Technology, 9202S), phosphorylated AKT (Ser473) (1:1000) (Cell Signaling Technology, 4060) or total AKT (1:1000) (Cell Signaling Technology, 9272). As a loading control, beta-actin (1:5000) (Sigma‒Aldrich, A5441) was used. The following day, the membrane was washed with TBS supplemented with 0.1% Tween-20 and incubated with the corresponding secondary antibody (1:5000) (polyclonal goat anti-rabbit IgG (H + L) (Invitrogen, 65–6120)) (polyclonal rabbit anti-mouse IgG (H + L) (Invitrogen, 61–6520)) for 1 h at room temperature. For visualization of the protein of interest, the membrane was covered with enhanced chemiluminescence (ECL) (Thermo Fisher Scientific, 34580) before chemiluminescence signals were developed via an imaging system (Bio-Rad, ChemiDoc imaging system) (Amoushahi and Lykke-Hartmann, 2021; Ataei-Nazari et al., 2022; Madsen et al., 2023; Madsen et al., 2024).

### Immunofluorescence of KGN cells

For determination of intracellular protein localization, IF was performed. The human granulosa-like cell line KGN was purchased from RIKEN Bioresource Centre (Tsukuba, Japan) and used for the experiment since it maintains the characteristics of ovarian cells. The KGN cells tested negative for *mycoplasma* before all the experiments were performed (Eurofins Genomics). KGN cells were seeded on glass slides (Hounisen, ISO 8037/1) placed in 6-well plates (STARSTEDT, 83.3920) in culture medium containing Dulbecco’s modified Eagle’s medium (DMEM) (Thermo Fisher Scientific, 31331–028) supplemented with 10% FBS (Gibco, A5209502) and 1% P/S (100 mg/mL and 50 ng/mL) (Thermo Fisher Scientific, 5140122). The following day, the cells were fixed in 4% PFA (Thermo Fisher Scientific, J199943-K2), permeabilized (3% BSA and 0.1% Triton X-100 in PBS) (Fluka BioChemika, 93418) and blocked (3% BSA in PBS) (HyClone, SH30574.02). The cells were subsequently incubated with CPS1 (1:200) (Abcam, 45956) as the primary antibody, followed by incubation with the secondary antibody Alexa Fluor 488 dye-donkey-anti-rabbit (1:300) (Thermo Fisher Scientific, A21206). Finally, counterstaining with DAPI (1:700) (1 μg/mL) (Thermo Fisher Scientific, 62248) was used, followed by mounting of the slides with fluorescence mounting medium. The cells were captured with an LSM800 laser-scanning confocal microscope.

### Arginine starvation assay

KGN cells were seeded at a density of 180,000 in a 6-well plate in culture media consisting of Dulbecco’s modified Eagle’s medium (DMEM) (Thermo Fisher Scientific, 31331–028) supplemented with 10% FBS (Gibco, A5209502) and 1% P/S (100 mg/mL and 50 ng/mL) (Thermo Fisher Scientific, 5140122) until 70% confluence. Next, the cells were cultured in DMEM lacking L-lysine and L-arginine (Fisher Scientific, 15728138) supplemented with L-lysine (0.0004986339 mol/L) (Merck, 4400-100 GM), 10% FBS and 1% P/S (100 mg/mL and 50 ng/mL) for 16 h. The following day, the cells were grown for an additional 2 h with DMEM supplemented with 10% FBS and 1% P/S or with DMEM lacking L-arginine supplemented with L-lysine, 10% FBS, 1% P/S and 30 µM NCG (1 mg/mL) (Sigma‒Aldrich, C4375-10G).

### Western blotting analysis of KGN cells

After the end of the culture period, the KGN cells were rinsed with PBS and lysed with RIPA buffer (Thermo Fisher Scientific, 89900) supplemented with 0.1% protease and phosphatase inhibitors (Thermo Fisher Scientific, 1861281). The protein concentration was measured by the Lowry method (Bio-Rad, 5000112) with BSA (HyClone, SH30574.02) as a standard (blank, 1.52, 0.76, 0.38, 0.19, 0.095 mg/mL), after which 25 µg of protein was used per lane. The samples were heated at 95 °C for 2 min before electrophoresis on SDS‒PAGE gels (Invitrogen, XV04120PK20), followed by transfer onto PVDF membranes (Thermo Fisher Scientific, 88518) for 1.5 h at 0.01 A. The membrane was blocked with Tris-buffered saline (pH = 7.6) (TBS) supplemented with 0.1% Tween-20% and 5% HyClone, SH30574.02) or 5% nonfat dry milk (MilliPore, 70166-500G) for 1 h at room temperature. The membranes were subsequently incubated overnight with primary antibodies against CPS1 (1:1000) (Abcam, ab45956), phosphorylated S6K (Thr389) (1:1000) (Cell Signaling Technology, 9205), total S6K (1:1000) (Cell Signaling Technology, 9202S), CASTOR1 (1:2000) (Thermo Fisher Scientific, PA5-78683) or MIOS (1:500) (Cell Signaling Technology, D12C6). As a loading control, beta-actin (1:5000) (Sigma‒Aldrich, A5441) was used. The following day, the membrane was washed with TBS supplemented with 0.1% Tween-20 and incubated with the corresponding secondary antibody (1:5000) polyclonal goat anti-rabbit IgG (H + L) (Invitrogen, 65–6120) (polyclonal rabbit anti-mouse IgG (H + L) (Invitrogen, 61–6520)) for 1 h at room temperature. For visualization of the protein of interest, the membrane was covered with enhanced chemiluminescence (ECL) (Thermo Fisher Scientific, 34580) before chemiluminescence signals were developed via an imaging system (Bio-Rad, ChemiDoc imaging system).

### pRK5 CASTOR1-HA cterm plasmid transfection of KGN cells

For analysis of the interaction between CASTOR1 and GATOR2, the pRK5 CASTOR1-HA Cterm plasmid purchased from David Sabatini (ddgene plasmid #84487; http://n2t.net/addgene:84487; RRID: Addgene_84487) was used for the transfection of KGN cells. The KGN cells were seeded in 6-well plates and grown in DMEM (Thermo Fisher Scientific, 31331–028) supplemented with 10% FBS (Gibco A5209502) and 1% P/S (100 mg/mL and 50 ng/mL) (Thermo Fisher Scientific, 5140122) until 70% confluence, after which the cells were transfected with the plasmid (2.5 µg/well) via Lipofectamine 3000 reagent (3.75 µL/well) as described by the manufacturer (Thermo Fisher Scientific, L300001). The cells were incubated at 37 °C for 24 h, after which the cells were arginine starved for 16 h as described in the “Arginine starvation assay” section. The cells were subsequently grown for 2 h in DMEM supplemented with 10% FBS and 1% P/S or in DMEM lacking arginine supplemented with 10% FBS, 1% P/S and 30 µM NCG (Sigma‒Aldrich, C4375-10G).

### Coimmunoprecipitation

For analysis of the interaction of the HA-tagged CASTOR1 protein with GATOR2 in KGN cells cultured in media supplemented with or without 30 µM NCG after arginine starvation, coimmunoprecipitation was performed using anti-HA magnetic beads as described by the manufacturer (Thermo Fisher Scientific, 88836). In brief, after transfection, arginine starvation and culture with or without 30 µM NCG, the cells were lysed in lysis buffer (25 mM Tris HCl (pH = 7.4), 150 mM NaCl, 1 mM EDTA, 1% Triton X-100, and 5% glycerol) supplemented with 1% protease and phosphatase inhibitors (Thermo Fisher Scientific, 1861281). The supernatant was incubated with anti-HA magnetic beads for 30 min, followed by washing. Finally, the bound protein was eluted with 1X sample buffer (6X: 0.3 M Tris, 25% glycerol, 8% SDS, 0.3 M DTT, bromophenol blue, pH = 6.8) by incubation at 95 °C for 10 min (Chantranupong et al., 2016).

#### siRNA transfection

In T25 flasks, 120,000 KGN cells were seeded and incubated at 37 °C in with an atmosphere of 95% humidity and 5% CO_2_ for 72 h. Hereafter, the KGN cells were transfected according to the manufacturer in DMEM supplemented with 10% FBS and either ON-TARGETplus Non-targeting control Pool (scramble siRNA) (Horizon Discovery, D-001810–10–05) or ON-TARGETplus Human CPS1 siRNA SMARTpool (CPS1 siRNA) (Horizon Discovery, L-009275-00–0005) and DharmaFECT 1 Transfection Reagent (Horizon Discovery, T-2001–03) as transfection reagent. The final concentration of the siRNA was 25 nM in each flask. The transfection media was removed 20 h later, and the cells were washed two times with PBS followed by incubation with DMEM supplemented with 10% FBS for 8 h. Next, the cells were cultured in either DMEM lacking L-lysine and L-arginine supplemented with L-lysine (0.0004986339 mol/L) and 10% FBS or normal DMEM supplemented with 10% FBS for 16 h. Next, the cells were grown for an additional 2 hours with DMEM supplemented with 10% FBS or with DMEM lacking L-arginine supplemented with L-lysine, 10% FBS, and 30 µM NCG (1 mg/mL).

#### eNOS activity assay

To determine the nitric oxide synthase (NOS) activity in KGN cells a colorimetric nitric oxide synthase activity assay kit was performed (Abcam, AB211083). In brief, KGN cells were grown in T175 flasks until 70% confluent, followed by 24 h grow with either DMEM supplemented with 10% FBS and 1% P/S or DMEM supplemented with 10% FBS, 1% P/S and 30 µM NCG (Sigma‒Aldrich, C4375-10G), followed by trypsinization and centrifugation. Hereafter, the NOS activity assay was performed according to the manufacturer. The absorbance was measured using a plate reader (BMG Labtech, CLARIOstar) at OD 540 nm, and the NOS specific activity was calculated according to the manufacturer.

## Statistics

All graphs and statistical analyses were completed with GraphPad Prism 10 (GraphPad Software, Inc., San Diego, CA, United States of America). The statistical results of all the experiments are presented as the mean values of all the replicates, with error bars indicating the standard error of the mean (SEM). The number of replicates in each experiment is stated in the results and visualized on the graphs.

To determine the statistical significance between groups with at least three replicates, an unpaired two-tailed *t*-test was used. p values <0.05 were considered significant. The assumption of normality was assessed with a QQ plot, and an *F* test was performed to test whether the observations came from the same distribution.

For statistical analysis of data with more than three datasets (histological analysis), one-way ANOVA was performed with a Bonferroni mean comparison. The normality of the data was assessed by QQ plots, and the assumption of equal homogeneity was validated via Bartlett’s test.

## Results

### The CPS1 protein is expressed in oocytes and granulosa cells from primordial, primary and secondary follicles

**FIGURE 1:**
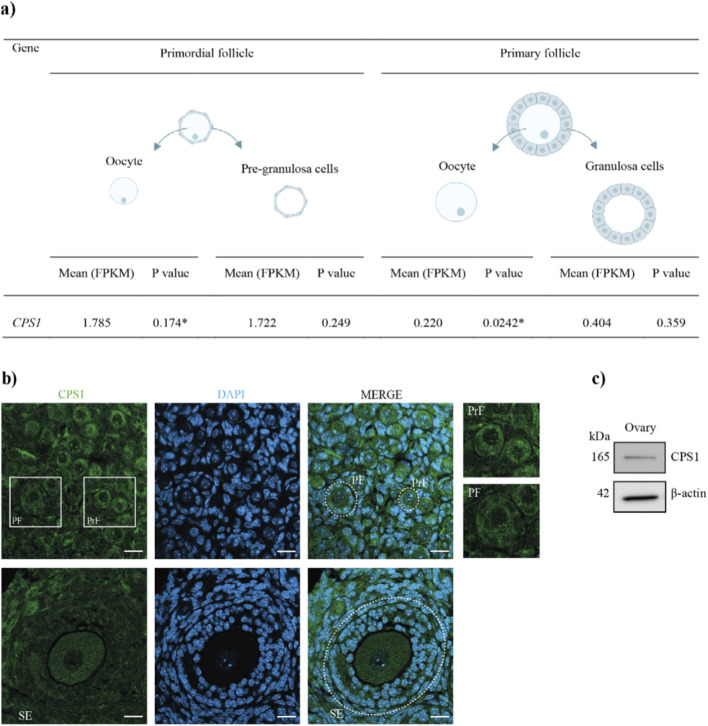
CPS1 expression in human and murine ovarian follicles. **(a)** The mean expression levels of *CPS1* in oocytes and granulosa cells from human primordial and primary follicles are shown (Ernst et al., 2018; Ernst et al., 2017). p values ≤0.2 were considered significant (*). **(b)** Immunofluorescence images of the distribution of the CPS1 protein in mouse ovaries (n = 3). The ovaries were cultured *in vitro* as controls. The left column shows CPS1, the middle column shows DAPI, and the right column shows a merged image of CPS1 and DAPI. The different follicle developmental stages are indicated: primordial (PrF), primary (PF), and secondary (SF). Scale bar: 20 μm, ×20 magnification. **(c)** A cropped version of the Western blotting analysis of CPS1 (full length in Supplementary Figure S1b). Three murine ovaries were pooled for each replicate (n = 3).

CPS1 is a ligase located in the mitochondria involved in the production of urea. We aimed to determine its role in primordial follicle regulation, and as a first step, we investigated the expression of the gene (*CPS1)* encoding CPS1 in ovarian follicles and the localization of the CPS1 protein in ovaries. To achieve this goal, we determined *CPS1* gene expression from transcriptomes of human oocytes and granulosa cells from primordial and primary follicles (Ernst et al., 2018; Ernst et al., 2017) to investigate in greater detail the intrafollicular distribution of the transcript. Gene expression was assessed according to the fragments per kilobase per million mapped fragments (FPKM), which is indicative of the mRNA level detected (Ernst et al., 2018; Ernst et al., 2017). The FPKM values ranged from 1 to 10 FPKM, where 1 is low expression and 10 is high expression (Ernst et al., 2018; Ernst et al., 2017). The RNA sequencing FPKM values indicated that the gene (*CPS1*) encoding the CPS1 protein ranged between 1.8 and 0.2 FRKM in oocytes and granulosa cells from primordial and primary follicles and was downregulated in primary follicles (Figure 1a). These findings suggest that the CPS1 transcript is expressed at relatively low levels in primordial follicles and is not expressed further in primary follicles. Despite weak detection of transcripts, the CPS1 protein might still be present in both follicle stages. Immunofluorescence was performed on murine ovarian sections to assess CPS1 protein distribution. An equal distribution of the CPS1 protein was observed in the oocytes and granulosa cells from primordial, primary and secondary follicles (Figure 1b; Supplementary Figure S1a). Western blotting confirmed a protein band corresponding to the molecular size of CPS1 in murine ovaries (Figure 1c; Supplementary Figure S1b). Although the CPS1 transcript was downregulated in human ovarian follicles, the CPS1 protein was at least detectable in murine ovaries, supported by the CPS1 protein expression noted in human ovaries (The Human Integrated Protein Expression Database (HIPED) (Schwanhausser et al., 2011), suggesting that the CPS1 protein is present during early follicle development.

### CPS1 activation promotes primordial follicle activation in murine ovaries

To investigate the potential role of CPS1 in primordial follicle regulation, we used NCG to induce CPS1 activation in *vitro*-cultured murine ovaries. Compared with the native activator NAG, NCG increases CPS1 activity with a greater km. Additionally, NCG is more resistant to degradation by cytosolic amino acylases, enabling it to reach the mitochondria more effectively (Chapel-Crespo et al., 2016).

**FIGURE 2:**
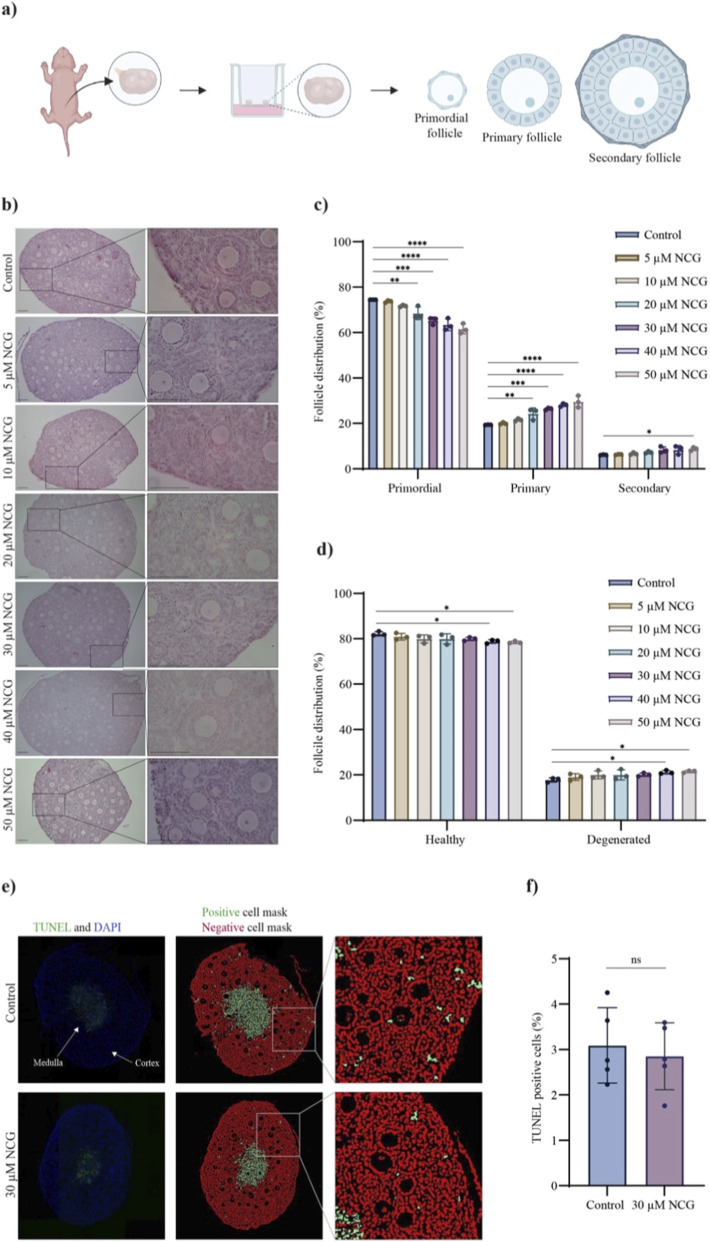
Histological analysis and apoptotic assay of follicle activation in *vitro*-cultured murine ovaries supplemented with or without NCG. **(a)** Schematic illustration of the method used for *in vitro* culture of 7–8-day-old murine ovaries. **(b)** Pictures showing a representative selection of H&E-stained mouse ovarian sections supplemented with different concentrations of NCG (0–50 µM) (n = 3). The first picture in each group shows one whole ovarian section (scale bar: 100 μm, ×10 magnification), and the black box illustrates the area for the close-up picture (scale bar: 100 μm, ×40 magnification). **(c)** Follicle distribution in *vitro-*cultured murine ovaries supplemented with or without NCG (0–50 µM) (n = 3). The follicle stages, primordial, primary, and secondary, are indicated on the x-axis, and the average percentage of follicles is indicated on the y-axis. The data are presented as the means±SEMs. One-way ANOVA was conducted, followed by multiple comparisons of the mean of each group to the mean of the control group. p values <0.05 = *, p values <0.01 = **, p values <0.001 = ***, p values <0.0001 = ****. (Follicle count in Supplementary Table S1) (Follicle distribution in Supplementary Table S2). **(d)** Images showing the distribution of healthy and degenerated follicles in *vitro-*cultured murine ovaries supplemented with or without NCG (0–50 µM) (n = 3). The data are presented as the means ± SEMs. One-way ANOVA was conducted, followed by multiple comparisons of the mean of each group to the mean of the control group. p values <0.05 = *. (Follicle count in Supplementary Table S3). **(e)** Representative repertoire of TUNEL-stained ovarian sections after *in vitro* culture with or without 30 µM NCG (n = 5). TUNEL and DAPI staining of the ovaries are shown in the first column. The arrows indicate the medulla and cortex of the ovaries. The second column shows ovarian sections where the positive cells (dead cells) are marked in green, and the negative cells are marked in red, as determined via a customized analysis program. The grey box displays the position of the ovarian cortex close-up shown in the third column. (Negative control in Supplementary Figure S2a). **(f)** A plot of the percentage of positive and negative cells in the ovarian cortex that underwent TUNEL in ovaries supplemented with or without 30 µM NCG (n = 5). The data are presented as the means ± SEMs. A *t*-test was conducted. p value >0.05 = ns. (Supplementary Figure S2b).

Ovaries were isolated from juvenile mice and cultured *in vitro* for 96 h in medium supplemented with 0–50 µM NCG (Figure 2a) to determine the potential effect of NCG. The number of follicles in the three different preantral phases, primordial, primary and secondary, was counted. Primordial follicles were classified as oocytes surrounded by one layer of flattened pregranulosa cells, primary follicles consisted of oocytes surrounded by one layer of cuboidal granulosa cells, and secondary follicles were classified as oocytes surrounded by more than one layer of cuboidal granulosa cells (Figure 2a).

Representative images of the ovarian sections revealed intact ovaries in all groups with healthy looking follicles, defined as follicles with an intact oocyte surrounded by an organized layer of intact granulosa cells (Figure 2b). Furthermore, the total number of follicles was consistent across all groups, verifying the activating effect of NCG (Supplementary Table S1). The classification and counting of the follicles revealed that NCG dose-dependently increased primordial follicle activation (Figure 2c; Supplementary Table S2). However, compared with the control group, the 40 μM and 50 µM NCG groups showed a significant increase in degenerated follicles (Figure 2d; Supplementary Table S3). These results suggest that 30 µM NCG is the concentration that significantly increases primordial follicle activation but does not significantly affect degenerated follicles. Therefore, an examination of apoptosis, a parameter for assessing ovarian follicle quality, after 30 µM NCG supplementation was performed via a TUNEL assay. In the ovary, most of the follicles are located in the cortex; therefore, the percentage of apoptotic cells was estimated in the ovarian cortex to evaluate the follicles after 96 h of culture with or without 30 µM NCG. Compared with those in the control ovaries, the proportions of apoptotic cells in the ovaries cultured with 30 µM NCG were not significantly different (Figures 2e,f; Supplementary Figure S2).

In summary, NCG can activate primordial follicles in a mouse *in vitro* culture assay, with an optimal effect without a significant increase in degenerated follicles at 30 µM NCG and without effect on the proportion of apoptotic cells. For the subsequent experiments, we used 30 µM NCG compared with the control.

### NCG significantly increased the phosphorylation of S6K in *vitro*-cultured ovaries

**FIGURE 3:**
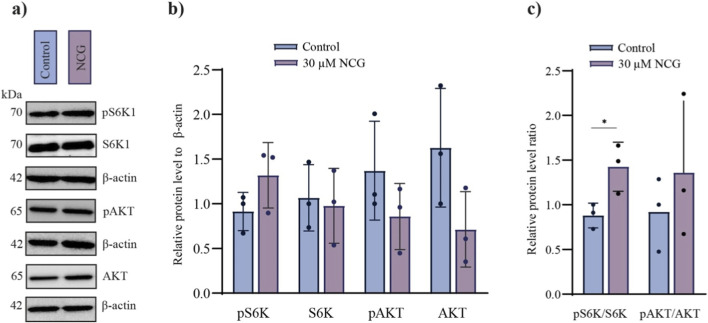
Levels of proteins known to be involved in primordial follicle activation in *vitro* cultured murine ovaries supplemented with or without 30 µM NCG. **(a)** A cropped version of the Western blotting analysis of pS6K1, S6K1, pAKT and AKT. Five murine ovaries cultured *in vitro* with or without 30 µM NCG were pooled for each replicate (n = 3). (Full length in Supplementary Figure S3). **(b)** A plot of the quantified Western blotting results. The protein name is indicated on the x-axis, and the protein normalized to β-actin is indicated on the y-axis. The data are presented as the mean ± SEM. A *t*-test was conducted. All p values were considered not significant (p value >0.05). **(c)** A plot of the ratio of active phosphorylated protein to total protein quantified via Western blotting analysis. The protein names are indicated on the x-axis, and the relative protein level ratio is indicated on the y-axis. The data are presented as the mean ± SEM. A *t*-test was conducted. p values <0.05 = *.

We next investigated whether NCG increased the activation of primordial follicles via the known PI3K/AKT and mTORC1 signalling pathways. *In vitro*-cultured murine ovaries were probed with primary antibodies against phosphorylated AKT and phosphorylated S6K1, as well as total AKT and S6K1, to determine the ratio of pAKT/AKT and pS6K/S6K. No significant difference was observed in the pAKT/AKT ratio normalized to β-actin when *in vitro*-cultured murine ovaries were supplemented with 30 µM NCG for 6 h. However, a statistically significant difference in the pS6K/S6K ratio normalized to β-actin was detected upon 30 µM NCG supplementation (Figures 3a-c; Supplementary Figure S3). These results indicate that the increase in primordial follicle activation observed with NCG supplementation is accompanied by activation of the mTORC1 signalling pathway.

### CPS1 activation by 30 µM NCG promotes the proliferation of KGN cells

**FIGURE 4:**
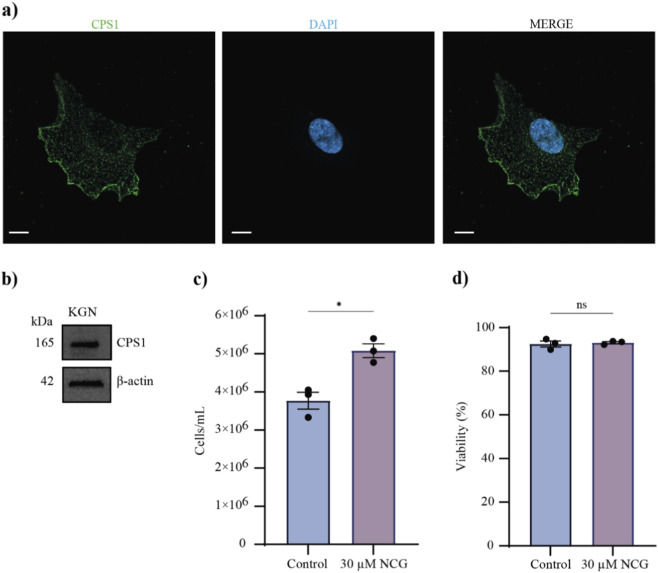
CPS1 expression in KGN cells and the effect of NCG on KGN cell proliferation. **(a)** Immunofluorescence images of the distribution of the CPS1 protein in KGN cells. The cells were cultured as controls. The left column shows CPS1, the middle column shows DAPI, and the right column shows a merged image of CPS1 and DAPI. Scale bar: 10 μm, ×63 magnification (Negative control in Supplementary Figure S4a). **(b)** A cropped version of the Western blotting analysis of CPS1. KGN cells cultured as controls were used (n = 4). (Full length in Supplementary Figure S4b). **(c)** A plot of the total number of cells after 24 h of culture with or without 30 µM NCG (n = 3). The data are presented as the means ± SEMs. A *t*-test was conducted. p values <0.05 = *. (Cell count in Supplementary Table S4). **(d)** A plot of the percentage of viable cells after 24 h of culture with or without 30 µM NCG (n = 3). The data are presented as the means ± SEMs. A *t*-test was conducted. p values >0.05 = ns.

To further investigate the molecular mechanisms underlying NCG activation of the mTORC1 signalling pathway, we used human granulosa-like tumour (KGN) cells. Immunofluorescence and Western blotting indicated that CPS1 is present in KGN cells (Figures 4a,b; Supplementary Figure S4), where the CPS1 protein was evenly distributed in the nucleus and cytoplasm of KGN cells (Figure 4a). When KGN cells were supplemented with 30 µM NCG for 24 h, a significant increase in cell count (Figure 4c; Supplementary Table S4) with no significant changes in viability (Figure 4d; Supplementary Table S4) was observed. These results are consistent with the activating effect of NCG observed in *vitro*-cultured murine ovaries and the function of mTOR as a protein kinase that controls cell proliferation.

### NCG supplementation of KGN cells led to an increase in arginine levels and the pS6K/S6K ratio

**FIGURE 5:**
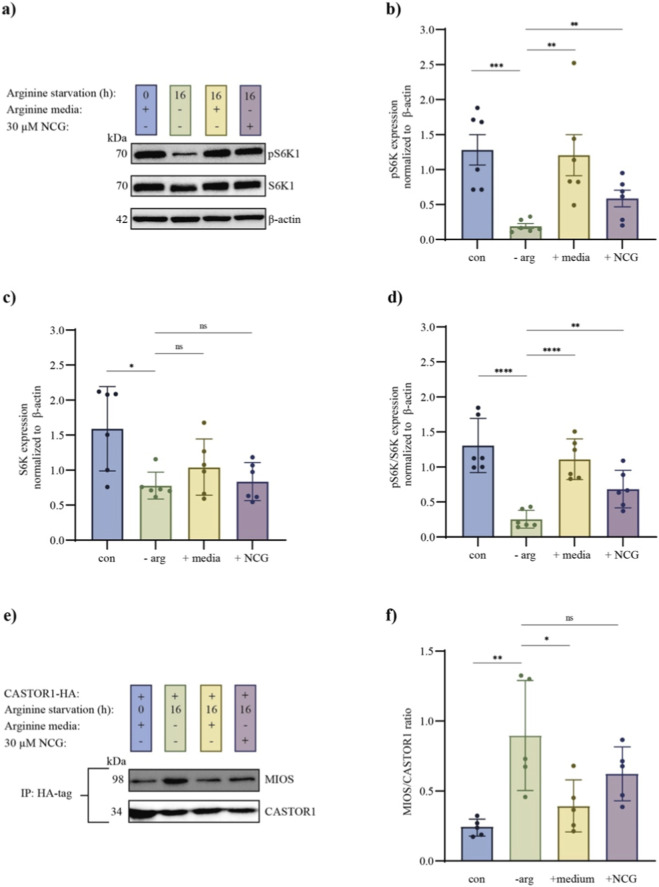
Effects of arginine starvation in KGN cells followed by 30 µM NCG supplementation on the binding of pS6K1, S6K and MIOS to CASTOR1. **(a)** A cropped version of the Western blottings of pS6K and total S6K. KGN cells were cultured until 70% confluence, followed by 18 h of culture in normal media or 16 h of arginine starvation and 2 h in media containing arginine- or arginine-free media supplemented with 30 µM NCG (n = 6) (full length in Supplementary Figure S7). **(b)** A plot of the quantified Western blotting of pS6K from **(a)**. The cell culture conditions are indicated on the x-axis, and pS6K normalized to β-actin is indicated on the y-axis. The data are presented as the mean ± SEM. A *t*-test was conducted. p values <0.01 = ** and p values <0.001 = ***. **(c)** A plot of the quantified Western blotting analysis of S6K from **(a)**. The cell culture conditions are indicated on the x-axis, and S6K normalized to β-actin is indicated on the y-axis. The data are presented as the mean ± SEM. A *t*-test was conducted. p values >0.05 = ns, and p values <0.05 = *. **(d)** A plot of the ratio of pS6K to total S6K quantified via Western blotting analysis. The cell culture conditions are indicated on the x-axis, and the relative protein level ratio is indicated on the y-axis. The data are presented as the mean ± SEM. A *t*-test was conducted. p values <0.01 = * and p values <0.0001 = ****. **(e)** A cropped image of the results of coimmunoprecipitation against the HA tag in KGN cells transfected with the pRK5 CASTOR1-HA Cterm plasmid for 24 h followed by different culture conditions as described in A (n = 5) (full length in Supplementary Figure S8). **(f)** A plot of the quantified coimmunoprecipitation data from **(a)**. The cell culture conditions are indicated on the x-axis, and the MIOS/CASTOR1 ratio is indicated on the y-axis. The data are presented as the mean ± SEM. A *t*-test was conducted. p values >0.05 = ns, p values <0.05 = * and p values <0.01 = **.

As a next step, we investigated whether the activating effect of NCG on mTORC1 was associated with an increase in arginine concentration. Arginine is a known upstream activator of mTORC1 signalling in other tissues, such as skeletal muscles (Yao et al., 2008), and human embryonic stem cells differentiate into fibroblasts, neurons and hepatocytes (Carroll et al., 2016). However, it remains unknown whether this mechanism occurs in ovarian cells. To clarify this issue, KGN cells were arginine starved for 16 h (time optimization of arginine starvation, Supplementary Figure S5), after which they were cultured in arginine-free media, media containing arginine or arginine-free media supplemented with NCG for 2 h (time optimization of NCG supplementation, Supplementary Figure S6). A positive control was generated without arginine starvation and cultured in media supplemented with arginine. Western blotting of pS6K, S6K and β-actin was performed to investigate the effect on pS6K (Figure 5a; Supplementary Figure S7) and revealed a significant difference in pS6K and S6K between the arginine starvation group and the control group (Figures 5b,c), leading to a significant difference in the pS6K/S6K ratio (Figure 5d). Furthermore, a significant increase in pS6K and the pS6K/S6K ratio was observed when NCG was added to arginine-starved cells; however, this increase was lower than that observed when media supplemented with arginine were added (Figures 5b-d).

To further address how NCG affects arginine levels in KGN cells, we performed coimmunoprecipitation of CASTOR1 and MIOS (subunit of the GATOR2 complex). GATOR2 and CASTOR1 binding is reduced when arginine is present in the cell, which leads to activation of the mTORC1 signalling pathway. KGN cells were transfected with the pRK5 CASTOR1-HA Cterm plasmid, which expresses CASTOR1 flanked with HA, the arginine sensor, upstream of mTORC1. Transfected KGN cells were arginine starved for 16 h before being cultured in arginine-free media, arginine-free media supplemented with 30 µM NCG or media supplemented with arginine for 2 h. A control without arginine starvation was cultured in media supplemented with arginine. Immunoprecipitation was performed against HA, and Western blotting was performed with primary antibodies against CASTOR1 and MIOS (Figure 5e; Supplementary Figure S8). Coimmunoprecipitation revealed that arginine starvation resulted in a significantly greater degree of binding between CASTOR1 and MIOS in KGN cells than in control cells (Figures 5e,f). A decrease in CASTOR1-MIOS binding was observed when KGN cells were cultured with media containing arginine or NCG after arginine starvation. This decrease in CASTOR1-MIOS binding was greater in media containing arginine than in those containing NCG. However, these observations were not statistically significant (Figures 5e,f).

**FIGURE 6:**
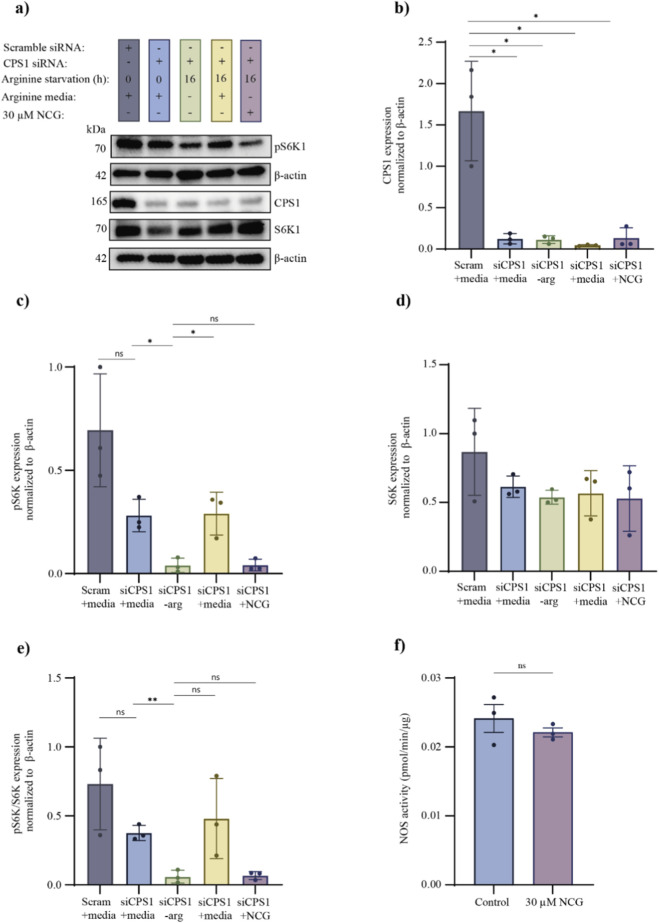
Effects of arginine starvation in KGN cells after siRNA-mediated knockdown to reduce CPS1 protein levels and NOS activity in KGN cells. **(a)** A cropped version of the Western blottings of pS6K, S6K and CPS1. KGN cells were transfected with siRNA for 20 h, followed by 8 h normal culture. Hereafter, they were cultured for 18 h in normal media or 16 h of arginine starvation and 2 h in media containing arginine- or arginine-free media supplemented with 30 µM NCG (n = 6) (full length in Supplementary Figures S9, S10). **(b)** A plot of the quantified Western blotting of CPS1 from A. The cell culture conditions are indicated on the x-axis, and CPS1 normalized to β-actin is indicated on the y-axis. The data are presented as the mean ± SEM. A *t*-test was conducted. p values <0.05 = *. **(c)** A plot of the quantified blot analysis of pS6K from A. The cell culture conditions are indicated on the x-axis, and pS6K normalized to β-actin is indicated on the y-axis. The data are presented as the mean ± SEM. A *t*-test was conducted. p values >0.05 = ns, and p values <0.05 = *. **(d)** A plot of the quantified Western blotting analysis of S6K from **(a)**. The cell culture conditions are indicated on the x-axis, and S6K normalized to β-actin is indicated on the y-axis. The data are presented as the mean ± SEM. A *t*-test was conducted. No significant difference between groups. **(e)** A plot of the ratio of pS6K to total S6K quantified via Western blotting analysis. The cell culture conditions are indicated on the x-axis, and the relative protein level ratio is indicated on the y-axis. The data are presented as the mean ± SEM. A *t*-test was conducted. p values >0.05 = ns, and p values <0.05 = *. **(f)** A plot of the NOS activity measured as pmol/min/µg protein in KGN cells after 24 h culture in media supplemented with or without 30 µM NCG. The treatment of the groups are indicated at the x-axis. The data are presented as the mean ± SEM. A *t*-test was conducted. p values >0.05 = ns.

To examine if the increase in pS6K and the pS6K/S6K ratio in arginine starved cells after NCG supplementation is specific to CPS1, we performed a siRNA-mediated knockdown to reduce CPS1 protein levels in KGN cells. After 20 h of transfection with scramble siRNA or *Cps1* siRNA the cells were arginine starved for 16 h, after which they were cultured in arginine-free media, media containing arginine or arginine-free media supplemented with NCG for 2 hours. A positive control was generated without arginine starvation and cultured in media supplemented with arginine. Western blotting of CPS1 revealed a successful knockdown of CPS1 (Figures 6a,b; Supplementary Figure S9) compared to the scramble group. Additionally, Western blotting of pS6K and S6K revealed that NCG could not increase the pS6K/S6K ratio after CPS1 knockdown, while supplementation with arginine could rescue and increase pS6K and the pS6K/S6K ratio (Figures 6a-e; Supplementary Figures S9, S10).

To further elucidate if the increase in arginine levels affects other arginine-utilizing pathways, we examined the nitric oxide synthase (NOS) activity, since a study showed that endothelial nitric oxide synthase/cyclic guanosine monophosphate/protein kinase G (eNOS/cGMP/PKG) pathway can promote primordial follicle activation (Zhao et al., 2020). We measured the eNOS activity in KGN cells after 24 h supplementation with 30 µM NCG and observed no significant difference between the groups (Figure 6f).

Together these results indicate that 30 µM NCG increased arginine concentrations, leading to activated mTORC1 and thereby phosphorylation of S6K. In the absence of arginine, CASTOR1 binds to GATOR2, inhibiting mTORC1 activation, and when arginine levels increase (due to 30 µM NCG treatment), CASTOR1 releases GATOR2. Free GATOR2 inhibits GATOR1, which is a negative regulator of mTORC1. Activated mTORC1 can phosphorylate downstream targets like S6K. When CPS1 was knocked down the phosphorylation of mTORC1’s downstream target S6K could not occur upon NCG supplementation, indicating that NCG increases arginine levels by activating CPS1. Additionally, eNOS activity was not altered despite an increase in intracellular arginine levels, suggesting that the effects of elevated arginine may be preferentially associated with activation of mTORC1 signalling rather than eNOS activation.

### *Ex vivo* culture of human ovarian tissue with NCG increased primordial follicle activation

**FIGURE 7:**
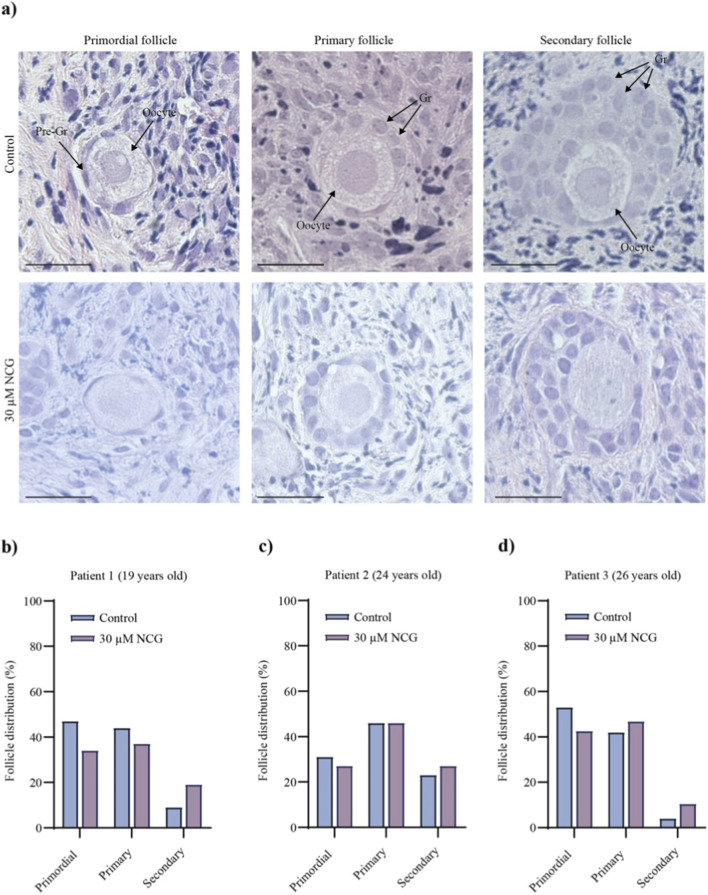
Histological analysis of follicle activation in *ex vivo* cultured human ovarian tissue supplemented with or without NCG. **(a)** Pictures showing representative primordial, primary and secondary follicles in H&E-stained human ovarian tissue from 14 days of *ex vivo* culture with or without 30 µM NCG (n = 18, n = 19, n = 22) (scale bar: 100 μm, ×40 magnification). **(b-d)** A plot of follicle distribution in *ex vivo-*cultured human ovarian tissue from three different patients supplemented with or without 30 µM NCG (n = 18, n = 19, n = 22). The follicle stages, primordial, primary, and secondary, are indicated on the x-axis, and the average percentage of follicles is indicated on the y-axis (follicle count in Supplementary Table S5).

As a next step, we investigated whether NCG could activate primordial follicles in human tissue samples from three patients. Cortical tissue pieces of human ovaries from three patients (19 years old, 24 years old and 26 years old) were fragmented into smaller pieces (1 × 1 mm) and cultured *ex vivo* for 14 days with or without 30 µM NCG (n = 18, n = 19, n = 22). After culture, the follicle distribution of primordial, primary and secondary follicles was examined (Figure 7a). Primordial follicles were classified as oocytes surrounded by one layer of flattened pregranulosa cells, primary follicles as oocytes surrounded by one layer of cuboidal granulosa cells, and secondary follicles as oocytes surrounded by more than one layer of cuboidal granulosa cells (Figure 7a).

The classification and counting of the follicles revealed a tendency for NCG to increase primordial follicle activation (Figures 7b-d; Supplementary Table S5), as indicated by the percentages of follicles in the three stages. In patients 1 and 3, the addition of NCG to the *ex vivo* culture suggested that there was increased activation of primordial follicles compared with that in the control group. In Patient 2, a greater proportion of the follicles were primary in the control group; hence, the difference between primordial and primary follicles was greater in the NCG-supplemented tissue. In all three patients, a greater proportion of secondary follicles was noted in the NCG-supplemented tissue than in the control tissue. These results indicate a tendency towards primordial follicle activation after NCG supplementation, which corresponds to the results observed in *vitro*-cultured murine ovaries.

## Discussion

Our results indicate that NCG increases follicle development through its ability to increase the activation of primordial follicles. We titrated the effects of NCG with increased arginine concentrations, leading to less binding between CASTOR1 and its inhibitor GATOR2, causing phosphorylation of S6K and activation of the mTORC1 signalling pathway.

The ability of NCG to increase endogenous arginine synthesis and modulate metabolic pathways has been extensively investigated. Its ability to influence cellular growth and development has made it a subject of interest in reproductive biology, where studies have demonstrated that NCG promotes ovarian follicular development (Ma et al., 2020; Zhou et al., 2022). Our findings suggest that NCG dose-dependently increases primordial follicle activation align with those of a study performed in chickens where dietary supplementation with NCG accelerated follicular growth, by increasing ovarian angiogenesis via the upregulation of vascular endothelial growth factor (VEGF) and NO production (Ma et al., 2020). Consistent with these findings, studies in yaks have highlighted the role of NCG in modulating cholesterol metabolism, leading to increased steroidogenesis and improved follicular growth (Zhou et al., 2022). To elucidate how NCG increases primordial follicle activation in *vitro-*cultured mouse ovaries, we demonstrated that NCG increased mTORC1’s downstream factor pS6K/S6K ratio after 6 h of NCG supplementation, whereas no effect on the pAKT/AKT ratio was observed. This highlights differences in the response of the two well-known AKT and mTORC1 regulatory pathways of the primordial follicle pool signalling pathways (Fortune, 2003). NCG is an analogue of NAG, which is the natural activator of CPS1 and promotes the urea cycle, increasing endogenous arginine production (Ah Mew et al., 2010; Barmore et al., 2024; Caldovic et al., 2010; Chapel-Crespo et al., 2016; Diez-Fernandez and Häberle, 2017). In our study, we demonstrated that arginine-starved KGN cells have a decreased level of the mTORC1 downstream factor pS6K. However, when arginine-starved cells were cultured with NCG, we observed an increase in pS6K. The same effect was observed when arginine was added to the media. Furthermore, siRNA-mediated knockdown to reduce CPS1 protein levels upon arginine starvation eliminated NCG’s effect on pS6K. Therefore, the increase in the pS6K/S6K ratio observed in *vitro-*cultured mouse ovaries indicates that NCG increases arginine levels, leading to activation of the mTORC1 signalling pathway and thereby primordial follicle activation. It has been reported that CASTOR1 is an arginine sensor upstream of mTORC1 (Chantranupong et al., 2016). When arginine is present in human embryonic kidney 293 cells, CASTOR1 is unable to bind GATOR2, which terminates its inhibition of the mTORC1 signaling pathway (Chantranupong et al., 2016). Despite extensive evidence that arginine regulates mTORC1 in other tissues, such as skeletal muscles (Yao et al., 2008), and that human embryonic stem cells differentiate into fibroblasts, neurons and hepatocytes (Carroll et al., 2016), our understanding of how NCG can regulate follicle growth is lacking, although the mTORC1 signaling pathway is critical in regulating the primordial follicle pool. However, this activation of mTORC1 is known to be regulated through growth factors (Guo and Yu, 2019). Our results correspond well with the known arginine regulation of mTORC1 signalling observed in other tissues and reveal that mTORC1 signaling in primordial follicle activation might also be regulated by arginine concentrations and not limited to growth factors. Although arginine regulation of mTORC1 has not been previously demonstrated in primordial follicle activation, a study investigating the effect of NCG in pregnant rats revealed that NCG supplementation elevated the serum levels of arginine, which promoted the activation of mTORC1 signaling. This increase promoted embryo implantation and increased the litter size (Zeng et al., 2012). In this study, trophoblast JAR cells were treated with arginine, which enhanced STAT3, PKB and S6K1 activation. These effects were abolished by pretreatment with an inhibitor of mammalian target of rapamycin (rapamycin).

NOS activity was included in the study to assess whether NCG potentially affects other arginine-utilizing pathways in the context of follicular activation. Nitric oxide (NO) has been implicated in ovarian physiology, and a study have suggested a role for eNOS/cGMP/PKG pathway in the regulation of primordial follicle activation (Zhao et al., 2020). However, despite elevated arginine levels, no change in NOS activity was observed in our study. This indicates that increased arginine levels alone are insufficient to modulate NOS in this context and does not support a role for NO-mediated mechanisms in the observed response. Instead, our findings are more consistent with arginine acting through arginine-sensing in the mTORC1 signaling.

The transcriptomic data from human oocytes and granulosa cells from primordial and primary follicles (Ernst et al., 2018; Ernst et al., 2017) revealed a decrease in the expression of CPS1 in oocytes from the primordial to primary follicle stages. However, our data revealed that the CPS1 protein is present, and thus, although *CPS1* transcript expression decreases, the CPS1 protein remains. During experiments with *in vitro*-cultured mouse ovaries, we could not distinguish NCG actions in oocytes and granulosa cells from those in the primordial and primary follicle stages. The ovary is a heterogenous organ composed of multiple cell types, and consequently the activation of mTORC1 signaling observed in the ovaries may be due to a response in the oocyte, granulosa cells or a combination of both. To partially address this limitation, and to elucidate the molecular mechanisms underlying the mTORC1-activating effect observed after NCG supplementation in murine ovaries, we used KGN cells as a model. Notably, the results obtained from KGN cells provide insight into only how NCG affects granulosa cells in the follicle and not the oocyte. Furthermore, KGN cells are altered granulosa cells with cancer signaling and thus do not represent both granulosa cells from primordial follicles and those from primary follicles, nor primary granulosa cells, thus, interpretation of the arginine-mTOR signaling in KGN cells might differ from physiological conditions and remains to be elucidated. However, since arginine regulation of mTORC1 is observed among different tissues, we would expect that this is also the mode of action in the oocyte upon NCG supplementation. Further studies are needed to distinguish between NCG contributions in oocytes and granulosa cells and how this interplay could regulate the activation of primordial follicles and support follicle growth. Studies addressing mechanisms acting in the oocyte versus those in granulosa cells during the earliest stages of follicle development (primordial and primary follicles), are based on transcriptome analysis ((Liu et al., 2024; Zhang et al., 2018), and a knock-out study (Takase et al., 2024) and these studies provide strong evidence that mechanisms in primordial and primary follicles can be experimentally and molecularly separated between oocyte and granulosa compartments, but are limited to descriptive conclusions.

Owing to the primordial follicle-activating effect observed in *vitro*-cultured murine ovaries supplemented with NCG, the translational aspect of NCG supplementation in humans is of interest. Therefore, we investigated the effects of NCG supplementation on *ex vivo* cultured fragments of human ovarian cortex from three different patients of reproductive age (19 years, 24 years, and 26 years). The results revealed that the primordial follicle activating effect of NCG supplementation observed in mouse ovaries has the same tendency in human tissue. This translational study is interesting because NCG is approved by the United States Food and Drug Administration and the European Medicines Agency, as it might assign a clinical aspect to NCG as a primordial follicle activator (Chapel-Crespo et al., 2016). However, the data is only included as a proof-of-concept study relying on three patients, and should be interpretated accordingly. There is no proof that systemic intake of NCG would have an effect on infertility, and neither dose nor side effects are currently reported. In chickens, increased plasma NO levels and upregulated expression of PKG-I, Raf1, and p-p38 increased angiogenesis in the ovaries. The chickens were fed dietary NCG (1%) for 14 days, which increased angiogenesis in the ovaries. To explore whether this phenomenon can be translated to human ovaries and represents a therapeutic option, further studies are needed to investigate the effects of NCG and CPS1 activation. Little research has focused on NCG and its breakdown products and potential side effects in ovaries. One important factor could be that the proliferation of ovarian somatic cells was promoted by NCG, whereas apoptosis was inhibited. A natural part of folliculogenesis and follicle selection is apoptosis, which promotes only one dominant follicle. Interference with this competition might have other concerns, which could complicate a clinical perspective.

Moreover, even though NCG has been used in clinical trials for hyperammonaemia, for patients with NAGS deficiency (Ah Mew et al., 2010; Chapel-Crespo et al., 2016; Diez-Fernandez and Häberle, 2017), it is unknown whether NCG could be feasible for ovarian therapies from a regulatory perspective.

## Conclusion

This study provides new insights into how NCG supplementation induces primordial follicle activation and how arginine also plays a role in primordial follicle activation through the mTORC1 signalling pathway, and future studies will determine the potential for primordial follicle activation from a clinical perspective. Using KGN cells (a human granulosa-like tumour cell line) versus ovary organ culture has several important limitations and considerations. KGN cells are cultured as a monolayer and lack the 3D structure and cellular diversity of the ovary. Moreover, KGN cells represent granulosa-like cells only whereas ovary organ culture includes multiple cell types (theca cells, oocytes, stromal cells), allowing for intercellular communication and more accurate modeling of ovarian function. KGN cells do not undergo folliculogenesis or oocyte maturation, in contrast to the ovary organ culture that allows for the study of follicle development.

## Data Availability

The original contributions presented in the study are included in the article/Supplementary Material, further inquiries can be directed to the corresponding author.
