# Supplementary material for: Activation of mitochondrial CPS1 promotes dormant ovarian follicle activation via arginine elevation and the mTORC1 pathway
Source: Front Cell Dev Biol. 2026 Apr 23;14:1787020. doi: 10.3389/fcell.2026.1787020 (PMC13149456; doi:10.3389/fcell.2026.1787020)
Supplement: Supplementary file 1 [file DataSheet1.pdf]

Supplementary Information

Supplementary Tables 1-5

Supplementary Figures 1-10

Table S1.

Follicle count in *in vitro* cultured murine ovaries supplemented with or without NCG. The total count of each follicle state (primordial, primary and secondary) in all ovaries cultured *in vitro* with or without NCG (0–50 µM) (n=3 in each group). The total follicle count for each ovary is indicated in the last column.

	**Follicle stages and counts**			
**N=3 for all groups**	**Primordial**	**Primary**	**Secondary**	**Total follicle count**
**Control**	1095	285	90	1470
	1005	260	85	1350
	920	240	75	1235
**5 µM NCG**	1140	305	100	1545
	975	260	80	1315
	1035	290	90	1415
**10 µM NCG**	800	235	80	1115
	1030	320	95	1445
	920	275	80	1275
**20 µM NCG**	940	280	90	1310
	825	315	95	1235
	840	325	90	1255
**30 µM NCG**	785	315	85	1185
	740	290	90	1120
	805	340	125	1270
**40 µM NCG**	690	310	100	1100
	690	285	65	1040
	745	350	120	1215
**50 µM NCG**	645	310	100	1055
	615	260	85	960
	607	365	90	1130

Table S2.

Follicle distribution in *in vitro* cultured murine ovaries supplemented with or without NCG. Statistical information on follicle distribution in murine ovaries cultured *in vitro* for 96 hours with or without NCG (0–50 μM) (n=3 in each group). One-way ANOVA was used to compare the differences in follicle distribution between the control group and the NCG groups. The p values are presented for each follicle stage. Multiple comparisons between the means of the control group and each NCG group were made according to the Bonferroni correction. Here, the mean values are presented ± the standard deviation (SD). p values > 0.05 = ns, p values < 0.05 = *, p values < 0.01 = **, p values < 0.001 = ***, p values < 0.0001 = ****.

	Mean (±SD) (%)							p values
**Treatment**	Control (n=3)	5 μM NCG (n=3)	10 μM NCG (n=3)	20 μM NCG (n=3)	30 μM NCG (n=3)	40 μM NCG (n=3)	50 μM NCG (n=3)	
Primordial follicles	74.48 (±0.027)	73.69 (±0.5065)	71.73 (±0.4386)	68.50 (±2.823) **	65.23 (±1.603) ***	1.603 (±2.594) ****	61.64 (±2.208) ****	<0.0001
Primary follicles	19.36 (±0.09021)	20.00 (±0.4265)	21.60 (±0.5351)	24.26 (±2.506) **	26.42 (±0.4625) ***	28.13 (±0.7028) ****	29.59 (±2.615) ****	<0.0001
Secondary follicles	6.164 (±0.1173)	6.306 (±0.2002)	6.675 (±0.4585)	7.245 (±0.4159)	8.350 (±1.362)	8.406 (±1.908)	(8.766) (±0.7609) *	ns

Table S3.

Healthy and degenerated follicles in *in vitro* cultured murine ovaries supplemented with or without NCG. Statistical information of the healthy and degenerated follicle distributions in murine ovaries cultured *in vitro* for 96 hours with or without NCG (0–50 µM) (n=3 in each group). One-way ANOVA was used to compare the differences between the control group and the NCG groups. The p values are presented. Multiple comparisons between the means of the control group and each NCG group were made according to the Bonferroni correction. Here, the mean values are presented ± the standard deviation (SD). p values > 0.05 = ns, p values < 0.05 = *.

	**Mean (±SD) (%)**							**p values**
**Treatment**	Control (n=3)	5 µM NCG (n=3)	10 µM NCG (n=3)	20 µM NCG (n=3)	30 µM NCG (n=3)	40 µM NCG (n=3)	50 µM NCG (n=3)	
Healthy follicles	82.22 (±0.9339)	80.94 (±1.452)	79.99 (±1.746)	79.96 (±2.233)	79.91 (±0.7460)	78.80 (±0.8058) *	78.45 (±0.4845) *	ns
Degenerated follicles	17.78 (±0.9339)	19.06 (±1.452)	20.01 (±1.746)	20.04 (±2.233)	20.09 (±0.7460)	21.20 (±0.8058) *	21.55 (±0.4845) *	ns

Table S4.

KGN cell count and viability with and without NCG treatment. KGN cells were counted, and viability was determined after 24 hours of growth with (n=3) or without 30 µM NCG (n=3).

	Mean (±SD)	
**Treatment**	Control (n=3)	30 µM NCG (n=3)
Cell count (cells/mL)	$3.77 \cdot 10^6$ ($3.86 \cdot 10^5$)	$5.08 \cdot 10^6$ ($3.15 \cdot 10^5$)
Viability (%)	92.47% (2.42%)	93.07% (85.05%)

Table S5.

Follicle distribution in *ex vivo* human tissue cultured with or without 30 µM NCG. The number of follicles in three different stages, namely, the primordial, primary and secondary stages, was determined in three different patients. The number of tissue pieces cultured in the control and NCG groups is indicated in the first column as n for each patient.

	Total count					
	Control			30 µM NCG		
	Primordial follicles	Primary follicles	Secondary follicles	Primordial follicles	Primary follicles	Secondary follicles
Patient 1 (19 years old) (n=18)	50	47	10	38	52	21
Patient 2 (24 years old) (n=19)	16	24	12	18	31	18
Patient 3 (26 years old) (n=22)	74	59	6	69	76	17

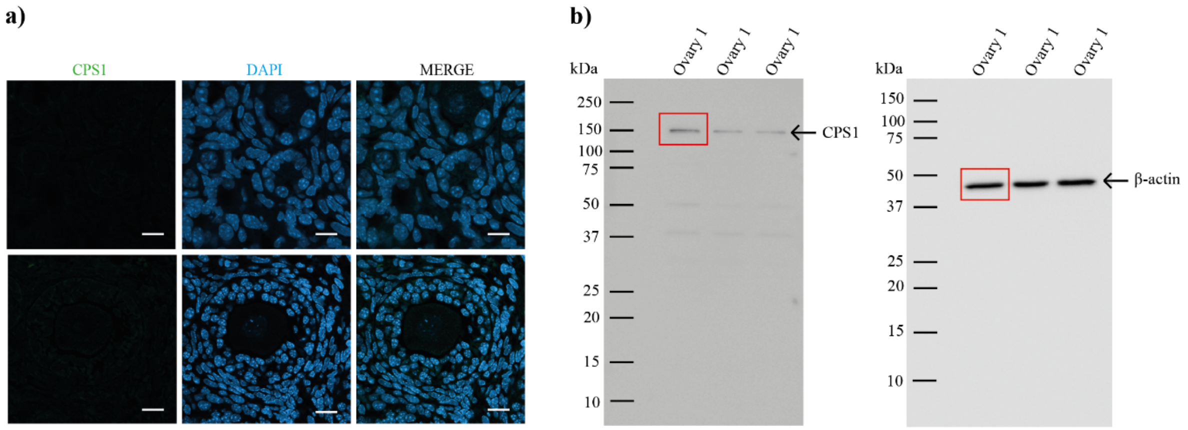

Fig. S1. Negative control of CPS1 immunofluorescence and full blot of CPS1 in murine ovaries. a) Immunofluorescence images of the negative control for CPS1 in murine ovaries (**Fig. 1b**). Scale bar: 20 µm, 20x magnification. **b)** Full Western blot analysis of CPS1 with β-actin as a loading control in murine ovaries (n=3). The red square indicates the bands used in **Fig. 1c**.

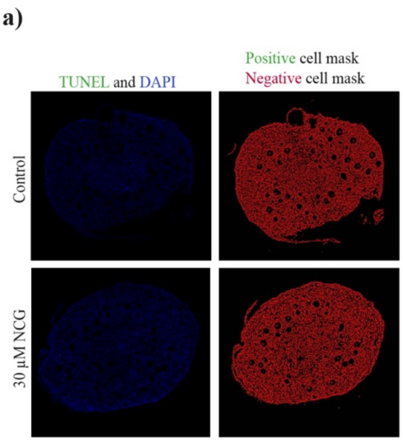

b)

	Mean (±SD) (%)	
**Treatment**	Control (n=5)	30 µM NCG (n=5)
TUNEL positive cells	3.091 (±0.8315)	2.853 (±0.7381)
TUNEL negative cells	96.91 (±0.8315)	97.15 (±0.7381)

Fig. S2. Negative control and statistical information of the TUNEL assay. A) Images of the negative control for TUNEL. The figure shows a representative repertoire of negative controls for TUNEL-stained ovarian sections after *in vitro* culture with or without 30 µM NCG for 96 hours (**Fig. 2e**). TUNEL staining of the ovaries, visualized with the FITC channel, and nuclear staining, visualized with the DAPI channel, are shown in the first column. The second column shows ovarian sections where the positive cells (dead cells) are marked in green, and the negative cells are marked in red, as determined via a customized analysis program. **B)** Statistical information of the percentage of positive and negative cells in the ovarian cortex that underwent TUNEL in ovaries supplemented with and without 30 µM NCG (n=5 for each group). The data are presented as the means ± SDs. A *t* test was conducted between the two groups. The p values were considered not significant (p value > 0.05) (**Fig. 2f**).

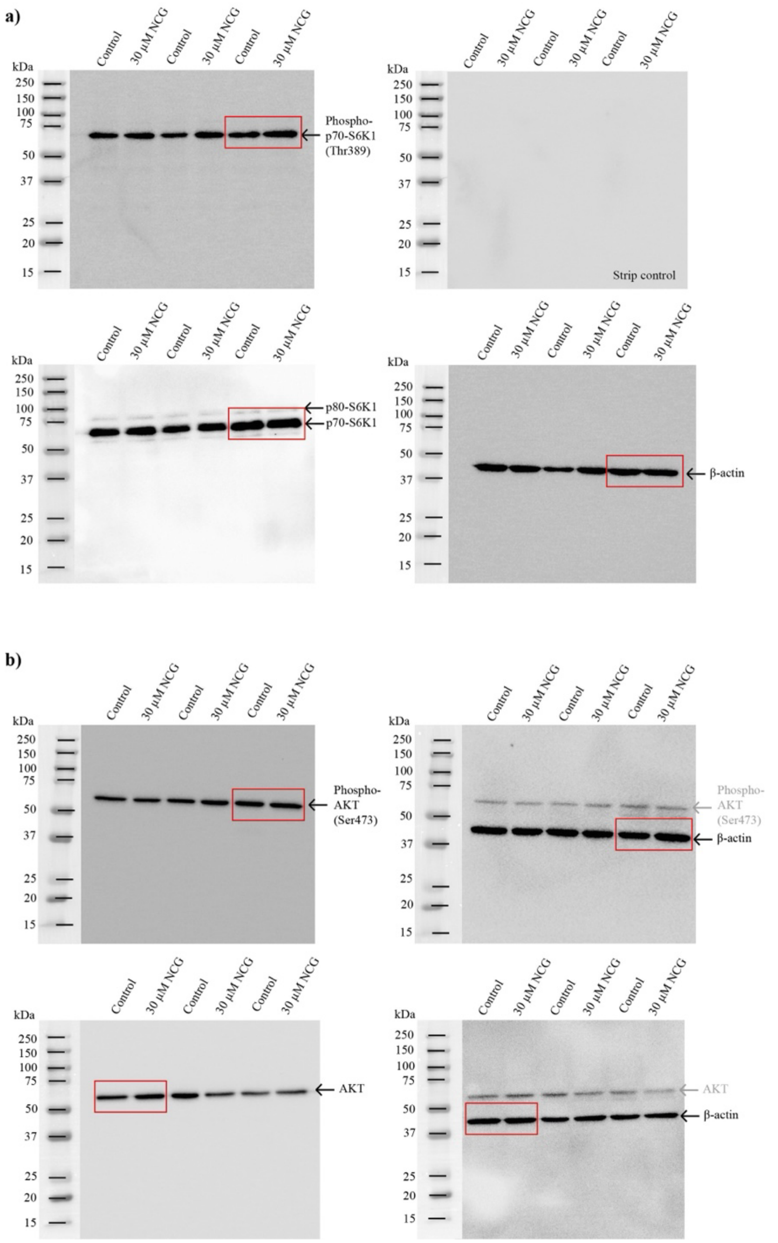

Fig. S3. Full blots of phospho-p70-S6K, p70-S6K, phospho-AKT and AKT in *in vitro*-cultured murine ovaries. a) Full Western blotting analysis of phosphor-p70-S6K, strip control, p70-S6K and β-actin as loading controls in 6-hour-old *in vitro*-cultured murine ovaries with or without 30 µM NCG. Five murine ovaries were pooled for each replicate (n=3). The red square indicates the bands used in **Fig. 3a**. **b)** Full Western blotting analysis of phosphor-AKT, strip control, AKT and β-actin as a loading control in 6 hour *in vitro*-cultured murine ovaries with or without 30 µM NCG. Five murine ovaries were pooled for each replicate (n=3). The red square indicates the bands used in **Fig. 3a**.

a)

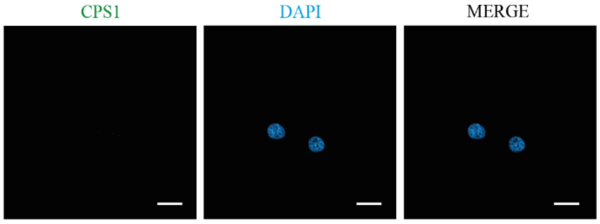

b)

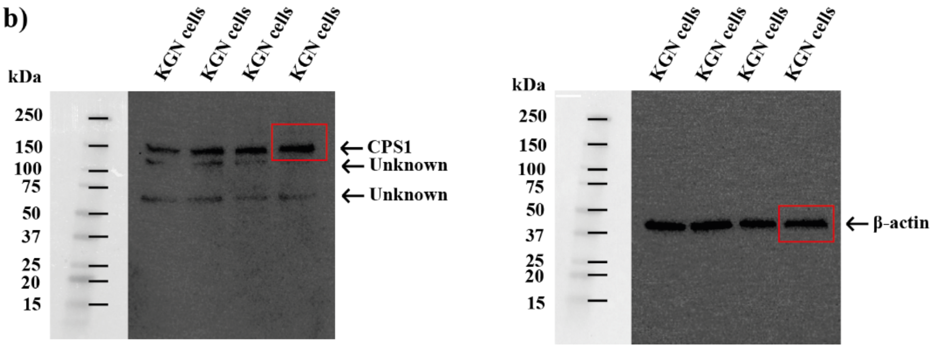

Fig. S4. Negative control for CPS1 immunofluorescence and full blot of CPS1 in KGN cells. a) Immunofluorescence images of the negative control for CPS1 in KGN cells (**Fig. 4a**). Scale bar: 10 µm, 20x magnification. **b)** Full Western blot of CPS1 with β-actin as a loading control in KGN cells (n=4). The red square indicates the bands used in **Fig. 4b**.

.

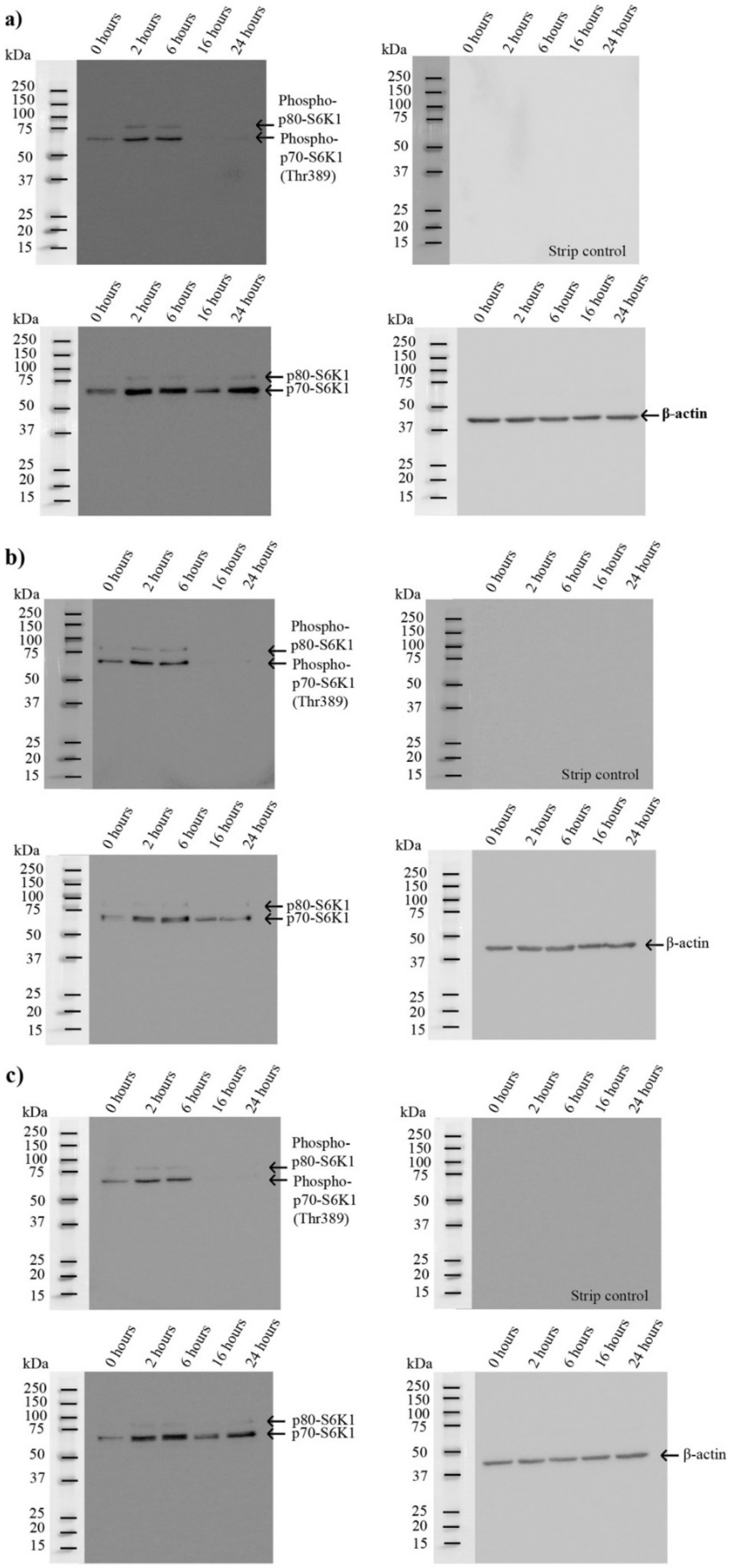

Fig. S5. Full blots of phospho-p70-S6K and p70-S6K for time optimization of arginine starvation in KGN cells. a, b, c) Full Western blot of phospho-p70-S6K, the strip control, p70-S6K and β-actin as a loading control in KGN cells after arginine starvation (n=3). The starvation time is indicated at the top of the blots.

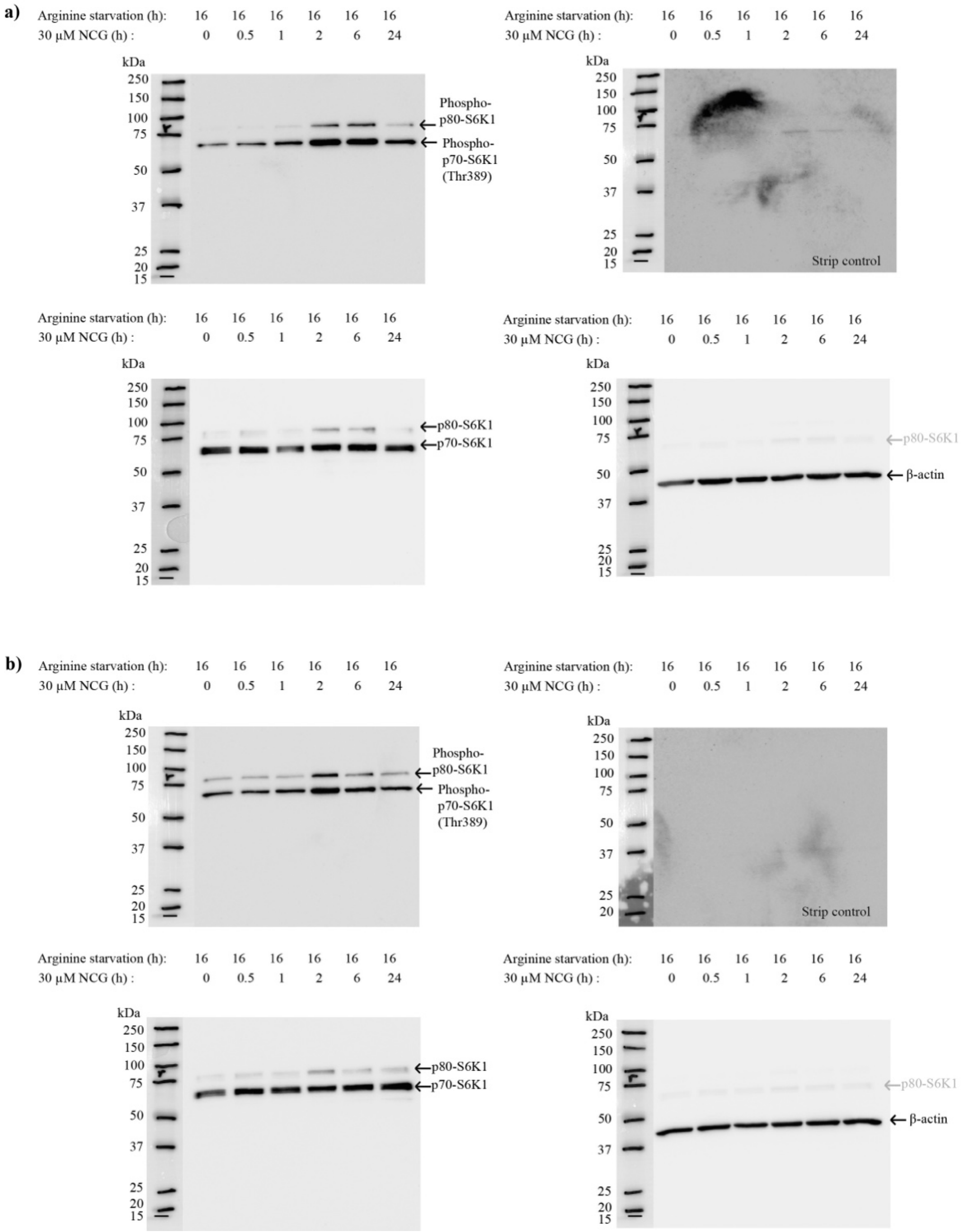

Fig. S6. Full blots of phospho-p70-S6K and p70-S6K for time optimization of NCG treatment in KGN cells. a, b) Full Western blot of phospho-p70-S6K, the strip control, p70-S6K and β-actin as a loading control in KGN cells after arginine starvation and supplementation with 30 µM NCG (n=2). The starvation time and the time for NCG supplementation are indicated at the top of the blots.

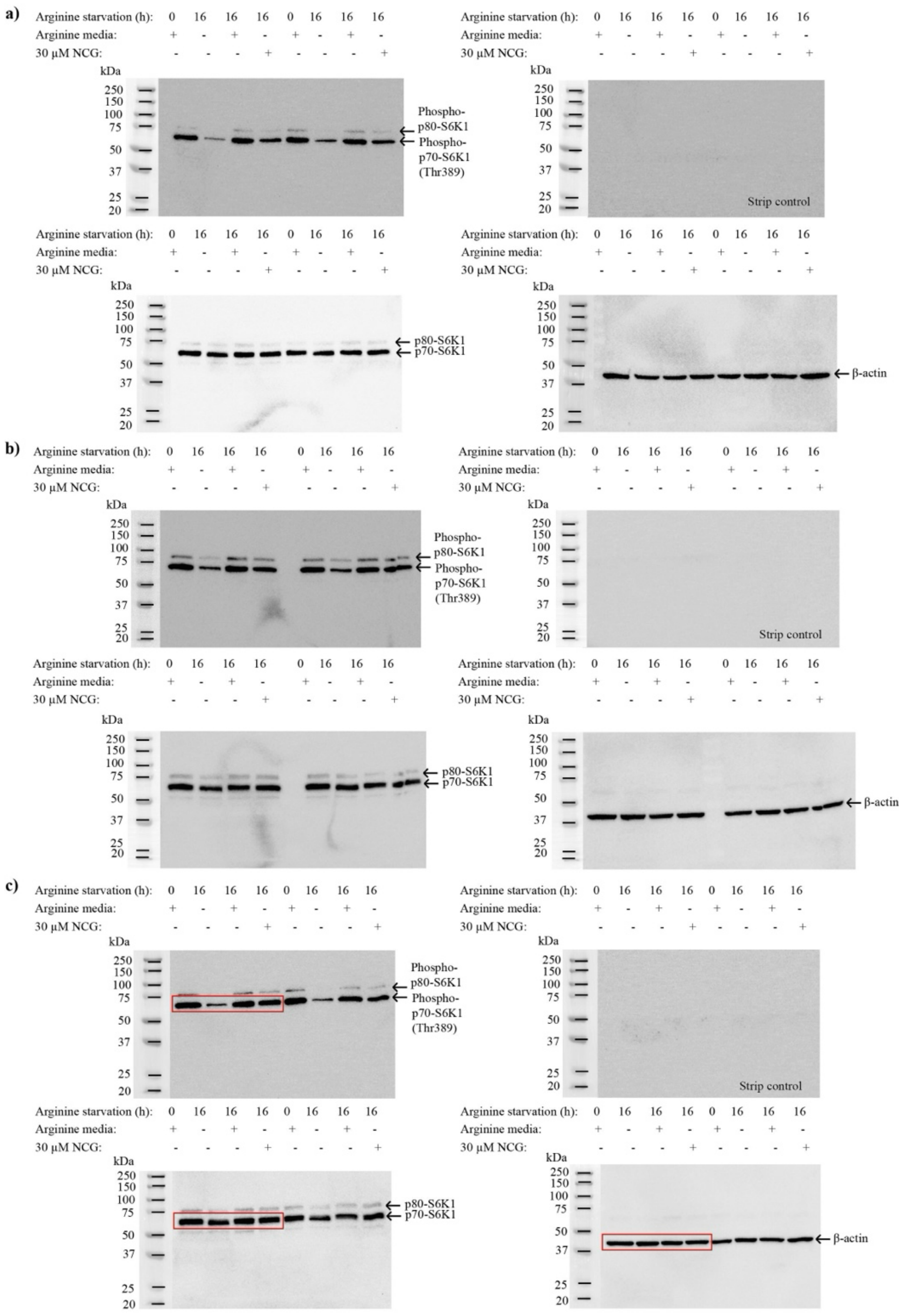

Fig. S7. Full blots of phospho-p70-S6K and p70-S6K KGN cells after arginine starvation and treatment. a, b, c) Full Western blot of phospho-p70-S6K, the strip control, p70-S6K and β-actin as a loading control in KGN cells after arginine starvation, followed by different treatments (n=6). At the top of each blot, the arginine starvation time is indicated. Furthermore, the following 2 hours of treatment with arginine media or 30 μM NCG are indicated +/- depending on the group. The red square marks the bands used in **Fig. 5a**.

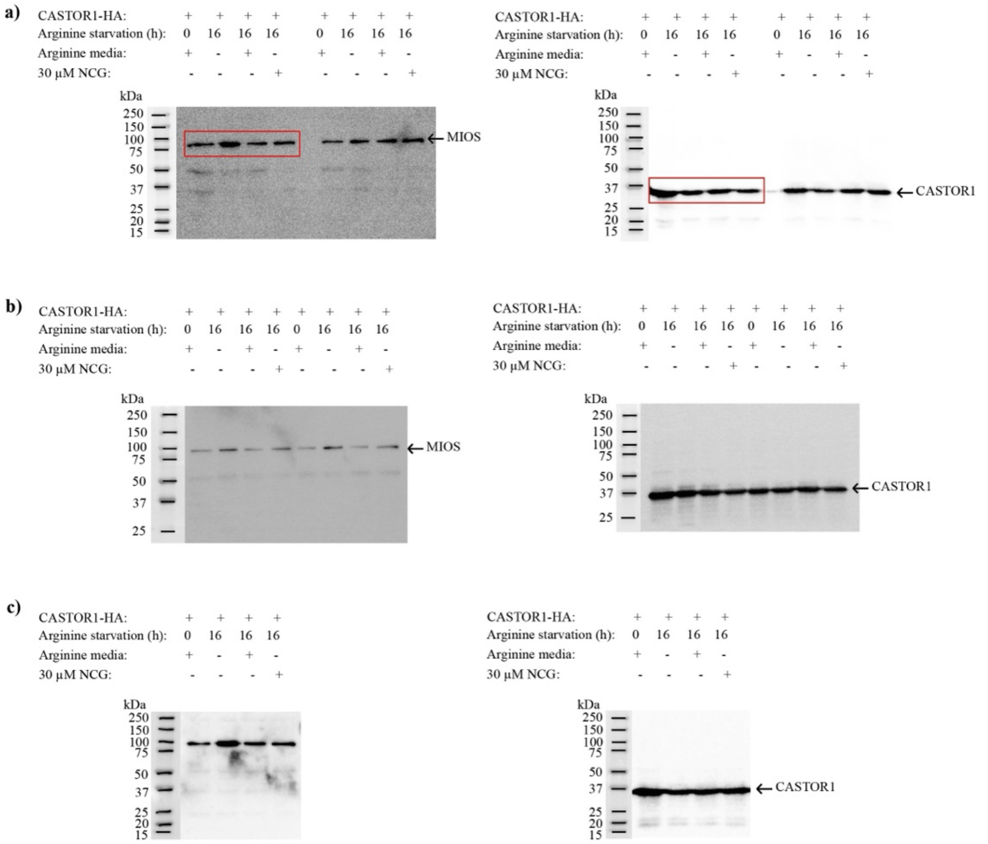

Fig. S8. Full blots of MIOS and CASTOR1 after coimmunoprecipitation in KGN cells. a, b, c) Full Western blots of CASTOR1 and MIOS after coimmunoprecipitation against the HA tag in KGN cells transfected with the pRK5 CASTOR1-HA Cterm plasmid for 24 hours followed by different culture conditions as indicated, with +/- in top of the blots. The red square marks the bands used in **Fig. 5e**.

a)

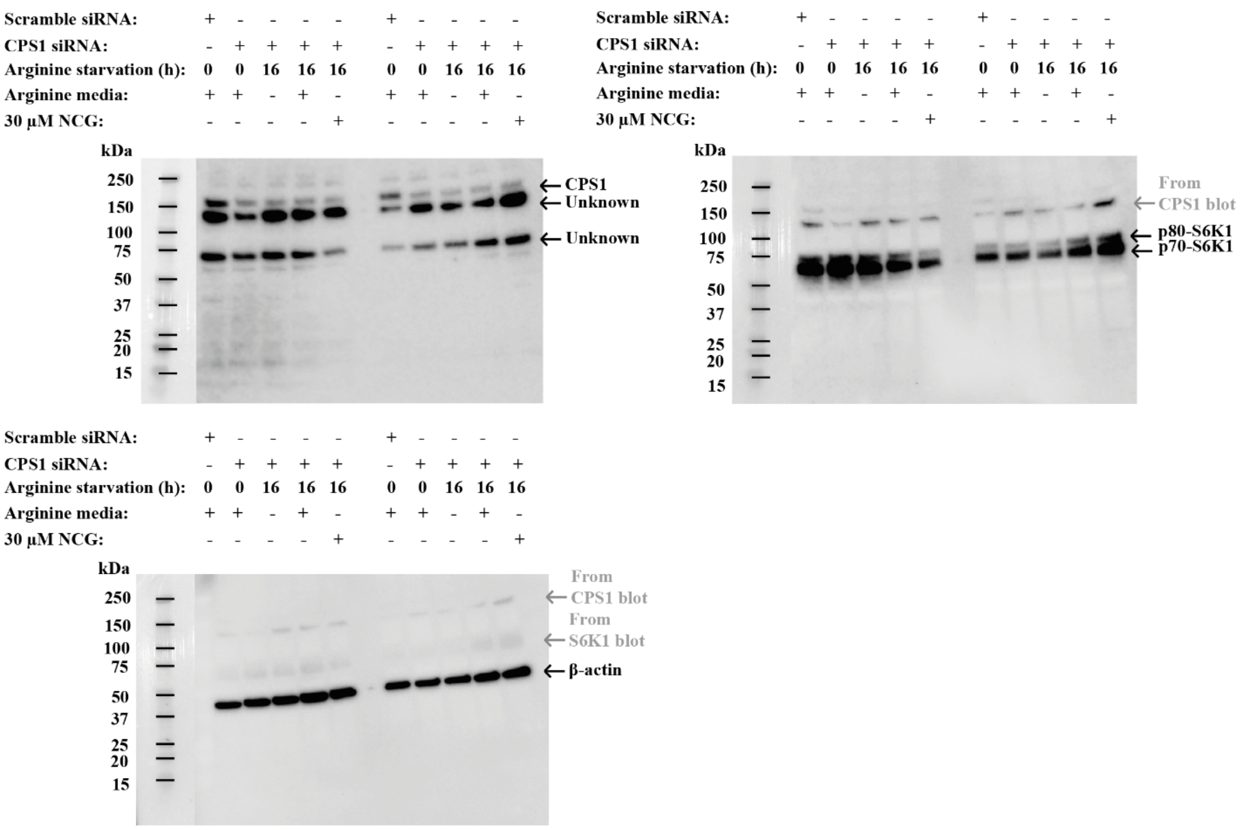
Scramble siRNA:
CPS1 siRNA:
Arginine starvation (h):
Arginine media:
30 µM NCG:
kDa
250
150
100
75
50
37
25
20
15
CPS1
Unknown
Unknown
From
CPS1 blot
p80-S6K1
p70-S6K1
From
S6K1 blot
β-actin

b)

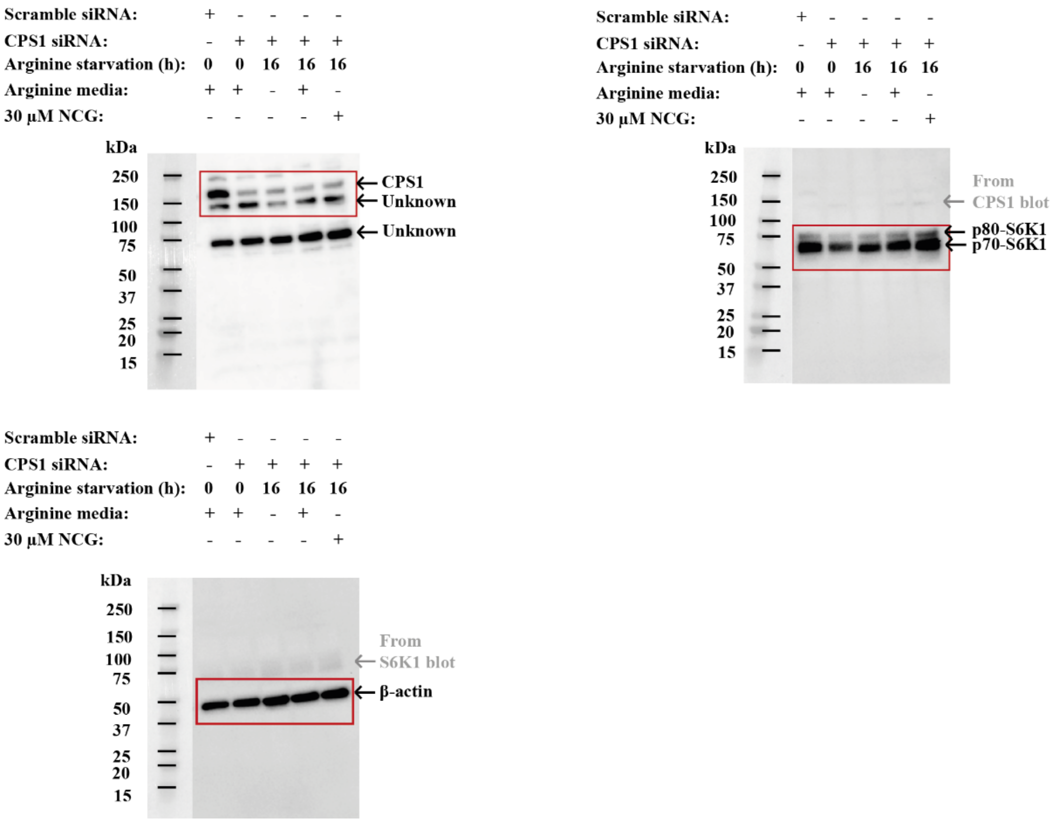
Scramble siRNA:
CPS1 siRNA:
Arginine starvation (h):
Arginine media:
30 µM NCG:
kDa
250
150
100
75
50
37
25
20
15
CPS1
Unknown
Unknown
From
CPS1 blot
p80-S6K1
p70-S6K1
From
S6K1 blot
β-actin

Fig. S9. Full blots of CPS1 and p70-S6K in KGN cells after siRNA transfection, arginine starvation and treatment. a, b) Full length Western blots of CPS1 and p70-S6K in KGN cells after siRNA transfection for 20 hours followed by different culture conditions as indicated, with +/- in top of the blots. The red square marks the bands used in **Fig. 6a**.

a)

b)

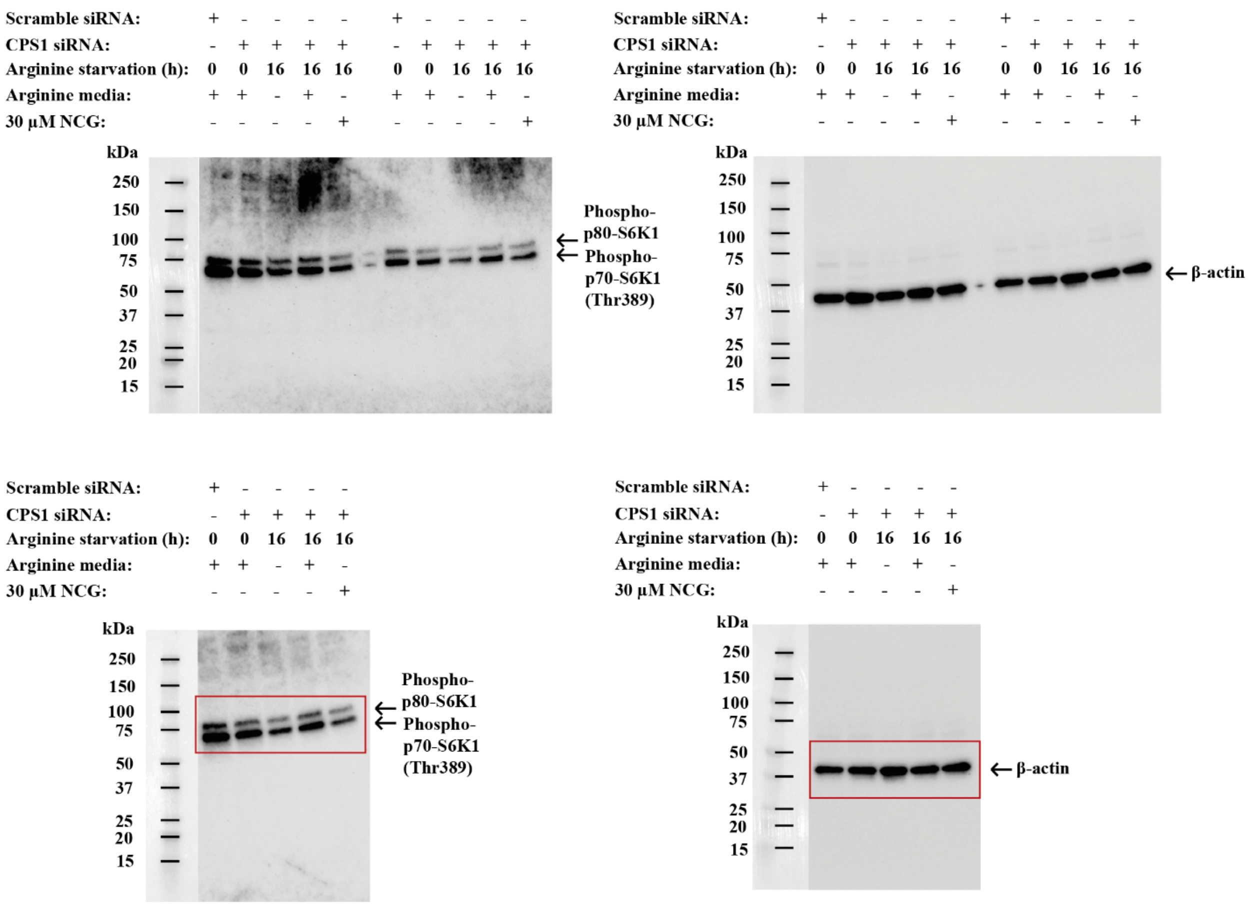

Fig. S10. Full blots of phospho-p70-S6K in KGN cells after siRNA transfection, arginine starvation and treatment. a, b) Full length Western blots of phosphor-p70-S6K in KGN cells after siRNA transfection for 20 hours followed by different culture conditions as indicated, with +/- in top of the blots. The red square marks the bands used in **Fig. 6a**.
